# 3D mapping of compositional gradients of core-shell structures in AgIn_*x*_Ga_1-*x*_S_2_ quantum dots by atom probe tomography

**DOI:** 10.1038/s41467-026-71518-5

**Published:** 2026-04-03

**Authors:** Byeong-Gyu Chae, Mihye Lim, Junho Lee, Nayoun Won, Soo Kyung Kwon, Ara Jo, Dong Jin Yun, Sangjun Lee, Jwa-Min Nam, Soohwan Sul, Tae-Gon Kim

**Affiliations:** 1 Analytical Engineering Group, Samsung Advanced Institute of Technology, Samsung Electronics Co., Ltd., Suwon, Republic of Korea; 2 Display Solution Platform, Samsung Advanced Institute of Technology, Samsung Electronics Co., Ltd., Suwon, Republic of Korea; 3 Department of Chemistry, Seoul National University, Seoul, Republic of Korea

**Keywords:** Quantum dots, Characterization and analytical techniques

## Abstract

Colloidal quantum dots (QDs), which exhibit tunable band gaps depending on their size and composition, are widely studied for light-emitting and optoelectronic applications. AgIn_*x*_Ga_1-*x*_S_2_-based QDs are particularly promising due to their pure green emission, high blue absorption, and environmental friendliness. However, a comprehensive understanding of these quaternary QDs remains challenging because of the difficulty in examining their complex compositional structure. Here, we three-dimensionally characterize quaternary QDs (AgIn_*x*_Ga_1-*x*_S_2_-based heterostructured QDs with core/shell and core/shell/shell structures) on the atomic scale using atom probe tomography. We reveal that both the AgIn_*x*_Ga_1-*x*_S_2_/AgGaS_2_ QDs with and without an outer ZnS shell have compositional gradients at their interfaces and elemental inhomogeneity among their cores. Furthermore, an Ag-deficient AgIn_*y*_Ga_1-*y*_S_2_ layer is identified on the outer surface of the AgGaS_2_ shell, where the stoichiometric fractions satisfy *x* » *y*, arising from differences in the precursor reactivity. Meanwhile, in the AgIn_*x*_Ga_1-*x*_S_2_/AgGaS_2_/ZnS QDs, the outer ZnS shell evolves into Zn_1-3/2*x*_Ga_*x*_S through a cation exchange process, ensuring structural and chemical compatibility with the inner shell. Our findings uncover the internal architecture and nanoscale elemental distributions of quaternary QDs, providing guidance for the future development of QDs.

## Abstract (web summary)

Core-shell structured quantum dots such as AgIn_*x*_Ga_1−*x*_S_2_ are promising materials for optoelectronics, but their compositional distribution remains poorly understood. Here, the authors characterize their structure at the atomic scale in 3D and reveal nanoscale compositional gradients.

## Introduction

Over the past several decades, colloidal semiconductor quantum dots (QDs) have garnered significant attention owing to their versatility in applications, including optoelectronic and biomedical devices^1–11^. QDs are particularly attractive because of their high photoluminescence (PL) performance, size-tunable optoelectronic characteristics, and cost-effectiveness^1–14^. The introduction of a core/shell structure, where the core is encapsulated by a shell, stabilizes QDs and increases their PL efficiency^1,6,9–11,14–17^. Recently, multinary I–III–VI-based QDs, such as AgInS_2_ and CuInS_2_, have emerged as promising environmentally friendly alternatives to conventional Cd- or In-based QDs for next-generation applications because their high compositional flexibility enables band-gap tuning^1,7,14–16,18–24^. Notably, the incorporation of Ga atoms into AgInS_2_ cores has produced green-emitting AgIn_*x*_Ga_1-*x*_S_2_-based QDs with enhanced color purity and strong blue absorption^18–24^.

Despite these advantages, AgIn_*x*_Ga_1-*x*_S_2_-based QDs still face critical challenges, including an inherently lower PL quantum yield (QY) and poor photochemical stability^20,21,24^. These issues may stem from factors such as the internal structure, compositional distribution, defects, elemental diffusion, and residual impurities. However, little is known about the atomistic mechanisms, such as the incorporation and diffusion of elements within AgIn_*x*_Ga_1-*x*_S_2_-based QDs. Several key questions remain unanswered: Is the QD structure a core/shell configuration? What is the three-dimensional (3D) compositional distribution within each layer? How do elements diffuse in heterostructured QDs? Are the interfaces and layers compositionally discrete or gradual? How do these elemental distributions affect the properties of QDs? What happens during shell growth on these complex structures?

Although the research on nanometer-sized core/shell QDs has advanced significantly through techniques such as transmission electron microscopy (TEM), X-ray photoelectron spectroscopy (XPS), and Fourier-transform infrared spectroscopy, these questions remain unresolved^25–30^. In particular, directly mapping the 3D distribution of constituent elements is a significant challenge. This difficulty might be due to the restricted spatial resolution and sensitivity of these instruments, as well as their inherent inability to analyze the 3D distribution in such complex structures. Atom probe tomography (APT) provides a potential solution by enabling direct 3D atomic imaging with a spatial resolution on the sub-nanometer scale^31–35^. In APT, an electric or laser pulse directed at a needle-shaped specimen ( < 100 nm apex) causes individual ions to evaporate and travel to a position-sensitive detector, where their time-of-flight yields the mass-to-charge ratio, enabling 3D elemental mapping^31–35^. However, applying APT to nanostructures, especially colloidal QDs, remains technically challenging because of their porosity, which complicates the preparation of needle-shaped specimens and often leads to structural instability.

In this study, we employ APT analysis to achieve the direct 3D mapping of two types of quaternary AgIn_*x*_Ga_1-*x*_S_2_-based heterostructured QDs: (1) AgIn_*x*_Ga_1-*x*_S_2_ core/AgGaS_2_ shell QDs and (2) AgIn_*x*_Ga_1-*x*_S_2_ core/AgGaS_2_ inner shell/ZnS outer shell QDs. The direct 3D observation of individual atoms enables us to uncover the complex internal structure of these QDs. We reveal that both types of QDs exhibit a compositional gradient across the layers and interfaces, rather than discrete boundaries. For the AgIn_*x*_Ga_1-*x*_S_2_/AgGaS_2_ QDs, we identify the formation of an Ag-deficient shell on the surface of the AgGaS_2_ shell during synthesis because of differences in the precursor reactivity. This Ag-deficient shell may sufficiently confine charge carriers while maintaining the crystalline structure. Interestingly, during the ZnS precursor step to form an outer shell on the AgIn_*x*_Ga_1-*x*_S_2_/AgGaS_2_ QDs, a Zn_1–3/2*x*_Ga_*x*_S outer shell spontaneously forms instead, driven by Ag^+^ and Ga^3+^ to Zn^2+^ cation exchange. This Zn_1–3/2*x*_Ga_*x*_S shell exhibits structural and chemical compatibility with the AgGaS_2_ inner shell and is expected to induce a Type-I band alignment. Additionally, APT analysis directly demonstrates noticeable compositional inhomogeneity among different AgIn_*x*_Ga_1-*x*_S_2_ cores, with off-stoichiometry in the core material. In addition to the effects of the size distribution, this nonuniform composition may also contribute to the inhomogeneous spectral broadening of the ultraviolet–visible (UV–Vis) absorption and PL band. These findings highlight the potential of APT to deepen our understanding of complex nanostructures and guide improvements in their performance.

## Results and discussion

### Structural and optical characteristics

**Fig. 1:**
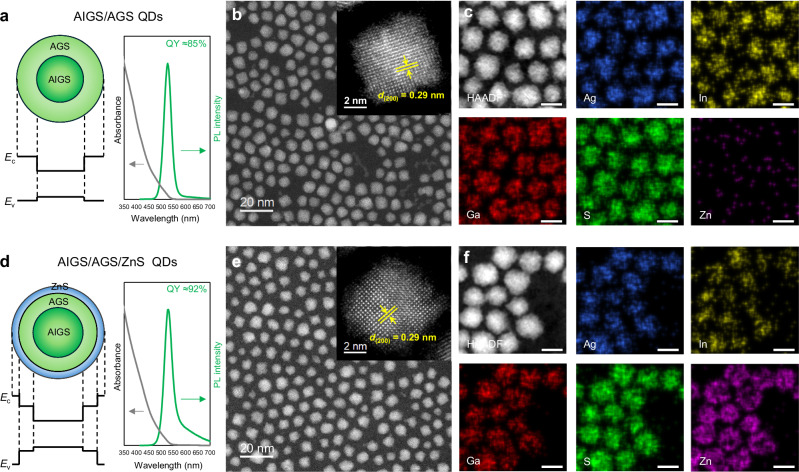
Comparison of AgIn_*x*_Ga_1-*x*_S_2_-based QDs before and after the ZnS precursor step. **a**, **d** Schematic representation of the internal structure, band diagrams, and UV–vis absorption, and PL characteristics of AgIn_*x*_Ga_1-*x*_S_2_/AgGaS_2_ (AIGS/AGS) and AgIn_*x*_Ga_1-*x*_S_2_/AgGaS_2_/ZnS (AIGS/AGS/ZnS) QDs. Both QDs exhibit bright green luminescence at ≈530 nm, with the QY increasing from 85% to 92% after the ZnS precursor step to form the outer shell. In addition, AgIn_*x*_Ga_1-*x*_S_2_/AgGaS_2_/ZnS QDs exhibit a long-wavelength emission tail in the ≈600–700 nm range. *E*_c_ and *E*_v_ denote the conduction band minimum and valence band maximum, respectively. **b**, **e** HAADF–STEM image of AgIn_*x*_Ga_1-*x*_S_2_-based QDs before and after the ZnS precursor step, which negligibly changed the size of the QDs, with average diameters of 6.14 ± 0.76 and 6.11 ± 1.03 nm, respectively. Size analysis was performed on more than 300 randomly selected QDs (*n* > 300). Inset: high-magnification HAADF–STEM image. Similar lattice fringes were consistently observed across multiple particles. **c**, **f** STEM–EDS elemental maps of AgIn_*x*_Ga_1-*x*_S_2_-based QDs before and after the ZnS precursor step. Colors: Ag, blue; In, yellow; Ga, red; S, green; Zn, violet. (Scale bars: 7 nm in (**c**, **f**)).

Figure 1a, d schematically illustrates the internal structure, band diagram, UV–Vis absorption, and PL characteristics of the AgIn_*x*_Ga_1-*x*_S_2_/AgGaS_2_ and AgIn_*x*_Ga_1-*x*_S_2_/AgGaS_2_/ZnS QDs, respectively. The AgIn_*x*_Ga_1-*x*_S_2_/AgGaS_2_ QDs were synthesized by modifying a method described in refs. ^18–20^. Both types of QDs exhibit bright green luminescence, with emission peaks at ≈530 nm. Notably, the QY increases from 85% to 92% after the ZnS precursor step. Furthermore, the stability of QD–acrylate composite films under ambient conditions was evaluated in terms of the change in the QY after 48 h with respect to the initial QY. As shown in Supplementary Fig. 1, the ZnS shell significantly improves this normalized QY from 66% to 97%. The enhanced optical properties can be attributed to the effective confinement of electrons and holes provided by the wide-bandgap ZnS encapsulating the AgIn_*x*_Ga_1-*x*_S_2_/AgGaS_2_. In addition to the dominant green emission, the AgIn_*x*_Ga_1-*x*_S_2_/AgGaS_2_/ZnS QDs exhibit a long-wavelength emission tail in the 600–700 nm range (Fig. 1d). The area ratio of this long-wavelength emission, defined as the fraction of the total PL intensity in the long-wavelength region (*λ* > *λ*_PL max_ + 50 nm), increases from ≈3.7% in AgIn_*x*_Ga_1-*x*_S_2_/AgGaS_2_ QDs to ≈14.9% in AgIn_*x*_Ga_1-*x*_S_2_/AgGaS_2_/ZnS QDs. Such long-wavelength emission is generally attributed to defect- or trap-assisted recombination rather than band-edge excitonic transitions in AgInS_2_-based QDs and related I–III–VI_2_-based QDs^36–40^.

The synthesized AgIn_*x*_Ga_1-*x*_S_2_/AgGaS_2_ core/shell and AgIn_*x*_Ga_1-*x*_S_2_/AgGaS_2_/ZnS core/shell/shell structured QDs were characterized using high-angle annular dark-field scanning transmission electron microscopy (HAADF–STEM). Figure 1b, e displays the HAADF–STEM images of the AgIn_*x*_Ga_1-*x*_S_2_/AgGaS_2_ QDs before and after the formation of the ZnS outer shell, respectively. The ZnS shell, which facilitates the formation of Type-I structures and improves the PL QY and stability, was synthesized through thermal decomposition^1,4,5,11,15,21^. The AgIn_*x*_Ga_1-*x*_S_2_/AgGaS_2_ QDs primarily exhibit a round-cornered polyhedral or spherical shape, with an average diameter of 6.14 ± 0.76 nm. Interestingly, after the ZnS precursor step for the outer shell formation, the QDs grow only negligibly, despite the addition of more precursors, and remain similar in both size and shape, with an average diameter of 6.11 ± 1.03 nm (Supplementary Fig. 2; see also Supplementary Fig. 3 for HAADF–STEM images of the core-only AgIn_*x*_Ga_1-*x*_S_2_ QDs). Moreover, the high-resolution STEM results reveal that the QDs are structurally similar before and after the ZnS precursor step, with all crystal planes showing a lattice spacing of ≈0.29 nm, which almost corresponds to the interplanar spacing of the (200) planes of chalcopyrite-structured AgGaS_2_. The lattice fringes are clearly evident not only inside the AgIn_*x*_Ga_1-*x*_S_2_-based QDs but also at the edges, indicating the high crystallinity across the entire QD structure (Fig. 1b, e). X-ray diffraction (XRD) measurements were further performed on the core-only AgIn_*x*_Ga_1-*x*_S_2_, AgIn_*x*_Ga_1-*x*_S_2_/AgGaS_2_, and AgIn_*x*_Ga_1-*x*_S_2_/AgGaS_2_/ZnS QDs to corroborate the structural similarity before and after shell formation. All XRD patterns are consistent with a chalcopyrite-based tetragonal structure, and the overall diffraction features remain essentially unchanged, even after the Zn precursor step (Supplementary Fig. 4). This comparative analysis indicates that the crystal structure is preserved throughout the shell formation process.

Meanwhile, HAADF–STEM imaging did not provide sufficient contrast to clearly distinguish the core from the shell. This limitation arises from the lack of significant mass differences between the constituent elements, as well as the similar crystallinity extending from the core to the shell. Specifically, for AgIn_*x*_Ga_1-*x*_S_2_-based QDs (*x* ≈ 0.5) synthesized for green emission in this study, the limited Z-contrast makes the core visually indistinguishable from the shell. The STEM–energy-dispersive X-ray spectroscopy (STEM–EDS) mapping showed that Zn atoms are distributed over a broader region than Ag and In after the Zn precursor step (Fig. 1c, f and Supplementary Fig. 5). However, the current STEM results remain insufficient for resolving the detailed internal and local compositional information of such 3D nanostructures. To identify the factors affecting the optical properties of the QDs and to further improve their performance, their internal structure must be more comprehensively understood.

### 3D compositional distribution of AgIn_*x*_Ga_1-*x*_S_2_/AgGaS_2_ QDs

To characterize and quantify the 3D atomic distribution of the AgIn_*x*_Ga_1-*x*_S_2_-based core/shell structured QDs, APT was employed. For the APT analysis in this work, dried QDs were prepared in powder form and analyzed following a method of ref. ^41^. In that study, we demonstrated that APT could resolve core/shell architectures and 3D compositional distributions in commercially available CdSe/ZnS core/shell QDs, in good agreement with TEM observations. Specimen preparation strategies, potential APT artifacts, and reconstruction considerations specific to CdSe/ZnS core/shell QDs were systematically examined, thereby establishing the applicability of APT to colloidal QD nanostructures. More broadly, the feasibility of APT in the analysis of metallic nanoparticles has been demonstrated in numerous prior studies, including ligand-resolved analyses and combined experiment–simulation approaches^42–47^. On this basis, we report a 3D atomic-scale characterization of AgIn_*x*_Ga_1-*x*_S_2_-based QDs using APT, providing detailed insight into their internal compositional structure.

**Fig. 2:**
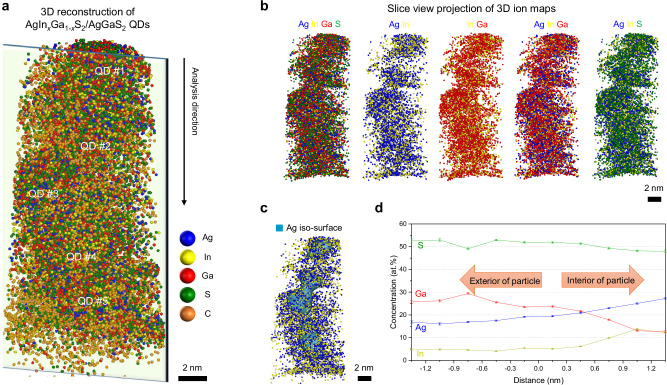
3D reconstruction and compositional analysis of AgIn_*x*_Ga_1-*x*_S_2_/AgGaS_2_ QDs using APT. **a** Reconstructed 3D atom map of the entire analyzed volume, showing five slightly ellipsoidal QDs. **b** Slice-view atom maps of Ag, In, Ga, and S, illustrating their internal spatial distributions, shown as 3 nm-thick slices. Ga and S atoms are more broadly distributed than Ag and In. **c** Ag iso-surface map and **d** proxigram showing 3D compositional trends; the proxigram was obtained from five QDs. The Ag and In contents increase toward the QD core, while the Ga content increases toward its outer surface, confirming the core/shell heterostructure. Colors: Ag, blue; In, yellow; Ga, red; S, green; C, orange. (Scale bars: 2 nm in (**a–c**)) Error bars in (**d**) indicate one-sigma counting statistics based on the detected ion counts within each sampling bin. The analysis is based on a single dataset without independent replicate measurements. Source data are provided as a Source Data file.

Figure 2a shows the 3D reconstructed APT atom maps of the AgIn_*x*_Ga_1-*x*_S_2_/AgGaS_2_ QDs, representing a hemispherical APT specimen containing five QDs (see Supplementary Fig. 6 for dashed guides indicating the approximate outlines of individual QDs). The QDs appear as slightly ellipsoidal features in the APT reconstruction, and a slice-view image along the *x*–*z* plane is presented in Fig. 2b to visualize their internal elemental distribution. We note that APT analyses of nanostructures may exhibit reconstruction artifacts, such as apparent elongation along the analysis (*z*) direction and interfacial broadening, primarily arising from local magnification and trajectory aberrations^42–52^. Accordingly, the slightly ellipsoidal morphology observed here is attributed to such effects rather than the intrinsic particle shape. However, compositional distributions along the *z*-direction have been shown to provide more reliable quantitative information than lateral directions because the reduced trajectory overlap suppresses artificial intermixing^42–44,51,52^. Under these conditions, compositional distributions and interfacial characteristics closer to the intrinsic distributions can be more evaluated.

APT shows that the detected Ga atoms are more broadly distributed than the Ag and In atoms in the QDs, and S atoms appear in both the core and shell regions of the QDs. Because the QDs are closely packed in a non-collinear configuration within the APT specimen and may therefore partially overlap in projection views, 3D reconstructions with C- and H-related ions removed are also provided in Supplementary Fig. 7 to better visualize the individual QDs. The 3D concentration profile obtained from an iso-concentration surface (proxigram) for Ag atoms reveals the average compositional trends across the five QDs (Fig. 2c). This profile more clearly indicates a higher concentration of Ag and In atoms toward the QD core, whereas Ga atoms increase toward the outer shell, confirming the formation of an AgIn_*x*_Ga_1-*x*_S_2_ core/AgGaS_2_ shell heterostructure.

**Fig. 3:**
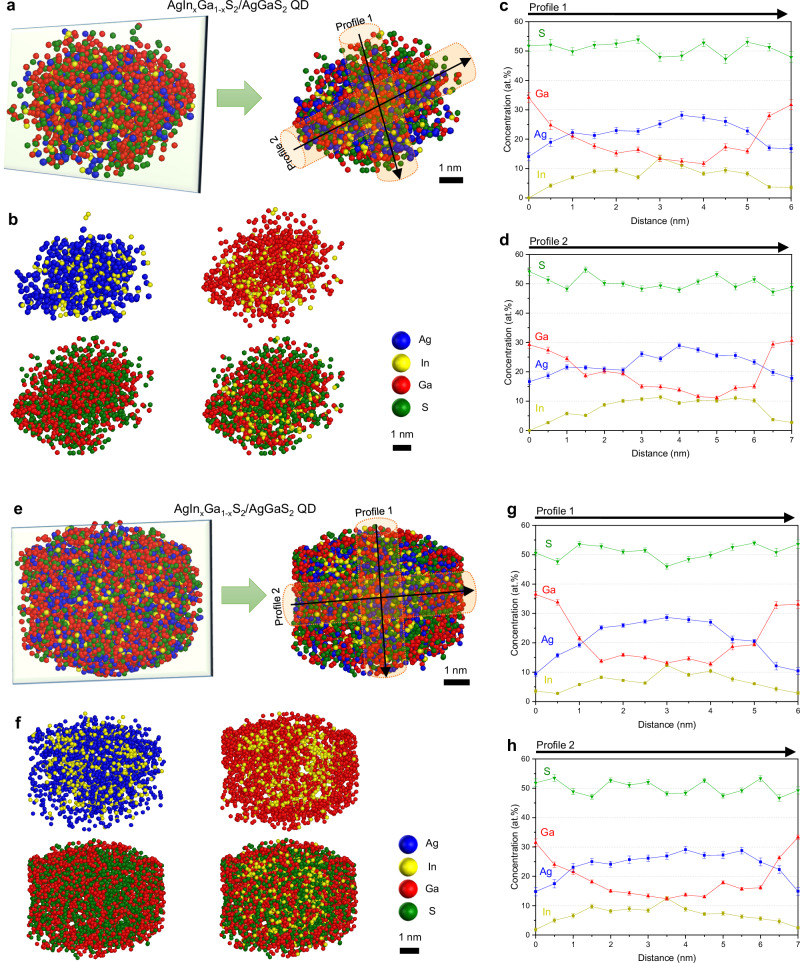
Compositional distributions of individual AgIn_x_Ga_1-x_S_2_/AgGaS_2_ QDs. **a**, **e** 3D atom map and cross-sectional atom map cropped by the green box. **b**, **f** Cross-sectional atom maps showing the spatial distribution of each element. Ga and S are broadly distributed, while Ag and In are present in small amounts in the shell regions. Colors: Ag, blue; In, yellow; Ga, red; S, green; C, orange. **c**, **d**, **g**, **h** 2D compositional profiles confirming the AgIn_*x*_Ga_1-*x*_S_2_ core/AgGaS_2_ shell structure and a compositional gradient. An Ag-deficient AgIn_*y*_Ga_1-*y*_S_2_ layer appears in the outermost region. (Scale bars: 1 nm in (**a**, **b**, **e**, **f**)) Error bars in (**c**, **d**, **g**, **h**) indicate one-sigma counting statistics based on the detected ion counts within each sampling bin. The analysis is based on a single dataset without independent replicate measurements. Source data are provided as a Source Data file.

To more precisely analyze the atomic-scale elemental distribution within individual QDs, two representative AgIn_*x*_Ga_1-*x*_S_2_/AgGaS_2_ QDs were extracted from the 3D reconstruction map. Figure 3a, d shows the 3D atom maps and the cross-sectional regions cropped by the green boxes for each QD. The cross-sectional maps of individual elements are presented in Fig. 3b, e, providing detailed insights into the internal structure. The 2D compositional profiles along two different directions (Fig. 3c, f) further confirm the formation of the AgIn_*x*_Ga_1-*x*_S_2_ core/AgGaS_2_ shell heterostructure with a compositional gradient. From the extracted profiles, we primarily focus on *z*-direction compositional analyses, cross-validated by proxigrams and lateral profiles. Ag and In atoms are highly concentrated at the center of the QD, at more than 20 and 10 at.%, respectively, but their concentrations gradually decrease toward the shell. Conversely, Ga atoms are less concentrated in the QD center but significantly more present in the outer shell. In addition, trace amounts of In atoms remain present in the shell region, leading to a shell composition closer to AgIn_y_Ga_1-y_S_2_, where *x* denotes the fraction of In in the core and *y* represents the residual In fraction in the outer layer, with *x* » *y*, rather than a pure AgGaS_2_ shell. This is likely due to the outward diffusion of In atoms from the AgIn_*x*_Ga_1-*x*_S_2_ core to vacant sites in AgGaS_2_, driven by the concentration gradient or thermal budget during synthesis. Nevertheless, the resulting AgIn_*y*_Ga_1-*y*_S_2_ shell is expected to effectively confine charges within the AgIn_*x*_Ga_1-*x*_S_2_ core owing to its higher band gap, consistent with a Type-I heterostructure, which promotes radiative recombination.

Another notable feature of the AgIn_*x*_Ga_1-*x*_S_2_/AgGaS_2_ QDs revealed by the APT analysis is the Ag-deficient outermost shell on the AgGaS_2_ shell, which, strictly speaking, corresponds to the AgIn_*y*_Ga_1-*y*_S_2_ shell. Comparing the cross-sectional atomic maps of Ag and Ga (Fig. 3b, e), Ga shows a broader distribution than Ag. The 2D compositional profiles also indicate a higher concentration of Ga atoms in the outermost region of the QDs, although distinguishing individual layers is challenging because of the compositional gradient (Fig. 3c, f). The unexpected formation of the Ag-deficient AgIn_*y*_Ga_1-*y*_S_2_ outermost shell is associated with the relatively lower reactivity of Ga^3+^ compared with that of Ag^+^ during the AgGaS_2_ shell synthesis. The lower reactivity between S^2−^ and group 13 elements, such as Ga^3+^, compared with that of group 11 elements, e.g., Ag^+^, has been observed when synthesizing group 11, 13, and 16 semiconductor QDs; hence, the reaction conditions must be precisely controlled to achieve the desired shell formation^53^. To compensate for the limited incorporation of Ga caused by its lower reactivity, we introduced an excess of Ga precursors during shell growth. Given that Ag^+^ is more reactive than Ga^3+^, Ag preferentially reacts with S during the initial stage of AgGaS_2_ shell growth. The excess Ga, which may not be readily incorporated during the early stage of the reaction, contributes to forming the Ag-deficient AgIn_*y*_Ga_1-*y*_S_2_ shell at a later stage. The resulting Ag-deficient outermost shell is expected to effectively passivate surface defects without introducing additional defect levels into the band gap, despite its Ag deficiency, thereby preserving the crystalline structure and stabilizing the QDs.

To demonstrate the role of differential precursor reactivity, we performed controlled syntheses in which the Ag precursor amount was increased at a fixed Ga level, a condition under which increasing the Ag precursor amount directly promotes the further growth of the AgGaS_2_ shell. STEM–EELS analyses showed the pronounced thickening of the AgGaS_2_ shell and consistently increased Ag incorporation, supporting the role of precursor reactivity in the formation of the shell (Supplementary Fig. 8 and Supplementary Table 1). These changes were accompanied by an increased contribution from trap-related emission and a decreased PL QY, indicating that Ag-driven shell growth directly impacts the optical properties of the QDs.

### 3D compositional distribution of AgIn_*x*_Ga_1-*x*_S_2_/AgGaS_2_/ZnS QDs

**Fig. 4:**
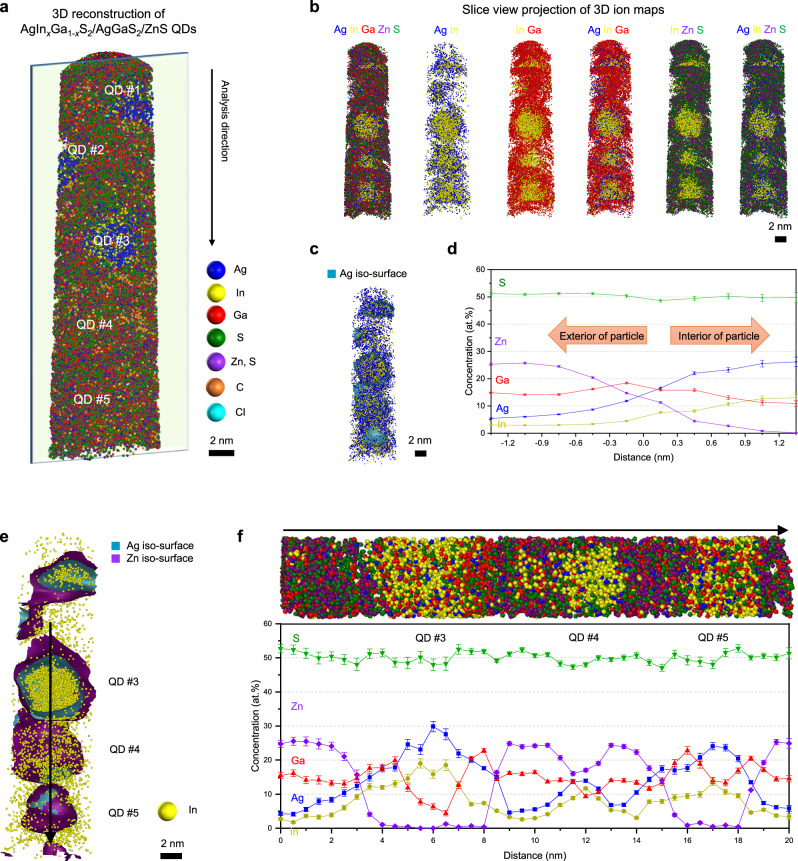
3D reconstruction and compositional analysis of AgIn_*x*_Ga_1-*x*_S_2_/AgGaS_2_/ZnS QDs using APT. **a** Reconstructed 3D atom map of the entire analyzed volume, showing five slightly ellipsoidal QDs. **b** Slice-view atom maps of Ag, In, Ga, Zn, and S, illustrating their internal spatial distributions, shown as 3 nm-thick slices. Although most Ag and In atoms are in the core regions, a significant amount of Ga is distributed over the shell region. Zn atoms predominantly exist in the outer shell. **c** Ag iso-surface and **d** proxigram obtained from Ag iso-surfaces; the proxigram was obtained from five QDs. The Ag and In contents increase toward the QD core, while the content of Zn increases toward the outer shell of the QD. A significant amount of Ga also appears in the outer shell. **e** Ag and Zn iso-surface and **f** 2D compositional profile extracted from a cylindrical volume (3 × 3 × 21 nm^3^) through the 3D reconstruction, confirming the core/shell/shell structure. Colors: Ag, blue; In, yellow; Ga, red; S, green; Zn, violet; C, orange; Cl, cyan. (Scale bars: 2 nm in (**a**–**c**, **e**)). The slight variations in the compositional distributions from QD to QD arise from the spatial offsets of individual QDs within the APT specimen. Error bars in (**d**, **f**) indicate one-sigma counting statistics based on the detected ion counts within each sampling bin. The analysis is based on a single dataset without independent replicate measurements. Source data are provided as a Source Data file.

Following the ZnS precursor step to form the outermost shell, APT was conducted to investigate the compositional evolution of AgIn_*x*_Ga_1-*x*_S_2_/AgGaS_2_/ZnS QDs. Figure 4a shows a reconstructed APT 3D atom map containing five nearly spherical AgIn_*x*_Ga_1-*x*_S_2_/AgGaS_2_/ZnS QDs (see Supplementary Fig. 9 for dashed guides indicating the approximate outlines of individual QDs). To further clarify the presence of these five QDs within the reconstructed volume and their internal structures, slice-view images along the *x*–*z* planes are provided in Fig. 4b. Although the 3D distribution of individual elements varies slightly among QDs depending on the cross-section position, all QDs consistently exhibit the AgIn_*x*_Ga_1-*x*_S_2_ core/AgGaS_2_ inner shell/Zn_1–3/2*x*_Ga_*x*_S outer shell structure, as shown in Fig. 4b. Further, the 3D atom maps show that Zn atoms, primarily located in the shell regions, are more broadly distributed than Ag and In. In APT, some Zn and S isotopes overlap in the mass spectrum. However, distinct peaks, such as S^+^, allow imaging with minimal interference from Zn isotopes (Supplementary Fig. 10). Interestingly, the Ga atoms, which are expected to reside primarily in the core and inner shell, are more broadly distributed throughout the QD than initially anticipated (Supplementary Fig. 11).

The proxigram from an iso-concentration surface of Ag atoms, which describes the five QDs, reveals that In atoms are more narrowly distributed than Ag atoms (Fig. 4c and Supplementary Fig. 12). Zn atoms are predominantly present in the outer shell, as evidenced by their increasing concentration toward the exterior. Ga atoms are primarily abundant in the AgGaS_2_ inner shell but are also present in significant amounts within the ZnS outer shell. The 2D concentration profile along the analysis direction offers additional insights into the internal structure of each QD (Fig. 4d). Although the elemental concentrations vary slightly depending on the profiling path owing to the spatial offset of individual QDs in the APT specimen, the 2D profile confirms the significant presence of Ga atoms in the outer shell, albeit at lower concentrations than Zn atoms. C-related signals, likely originating from ligands, were also observed on the outside of the QDs (Supplementary Fig. 13). Nevertheless, because of the difficulty in quantifying these signals, our discussion focuses on the distribution of the main elements (see Supplementary Fig. 14 for the 3D reconstructions with the C- and H-related ions removed for clarity). Notably, this work provides the first 3D atomic-scale insights into heterostructured quaternary QDs with a core/shell/shell geometry using APT.

**Fig. 5:**
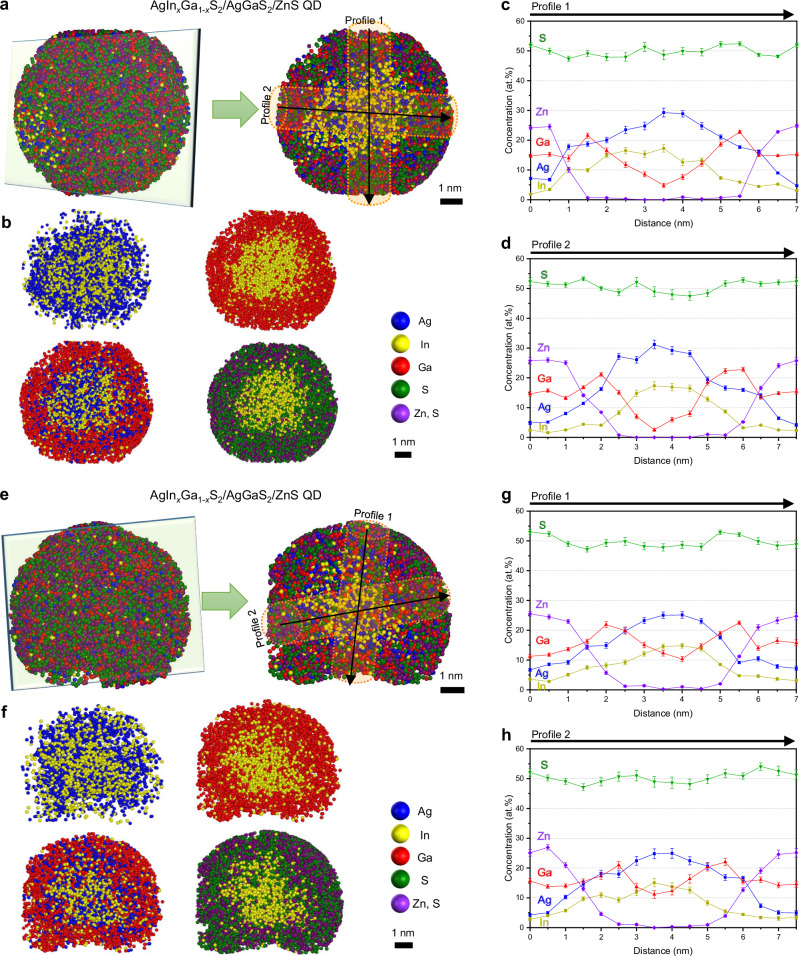
Compositional distributions of individual AgIn_*x*_Ga_1-*x*_S_2_/AgGaS_2_/ZnS QDs. **a**, **e** 3D atom map and cross-sectional atom map cropped by the green box. **b**, **f** Cross-sectional atom maps showing the spatial distribution of each element. Zn atoms are more broadly distributed than Ag and In. Ga atoms exhibit a broad distribution across the QDs, extending beyond the inner shell. Colors: Ag, blue; In, yellow; Ga, red; S, green; Zn, violet. **c**, **d**, **g**, **h** 2D compositional profiles confirming the AgIn_*x*_Ga_1-*x*_S_2_ core/AgGaS_2_ inner shell/ZnS outer shell structure with a gradual compositional gradient. Ga is observed in all layers, including the outer shell, which thus comprises Zn_1–3/2*x*_Ga_*x*_S rather than ZnS. Compositional differences between individual QDs, particularly in the core region, are also evident. (Scale bars: 1 nm in (**a**, **b**, **e**, **f**)) Error bars in (**c**, **d**, **g**, **h**) indicate one-sigma counting statistics based on the detected ion counts within each sampling bin. The analysis is based on a single dataset without independent replicate measurements. Source data are provided as a Source Data file.

To validate the internal structure and elemental distribution of AgIn_*x*_Ga_1-*x*_S_2_/AgGaS_2_/ZnS QDs, two representative QDs were extracted from the 3D reconstruction map (Fig. 5). Figure 5a, d shows the extracted 3D atom maps and the cross-sectional regions cropped by the green boxes for each QD. According to the *z*-direction compositional profiles cross-validated by proxigrams and lateral profiles, Ag atoms are more widely distributed than In atoms in the 3D atom maps (Fig. 5b, e). However, both Ag and In atoms are at their highest concentrations in the central region of the QDs, with values exceeding 20 and 10 at.%, respectively. The concentration of In atoms remains lower than that of Ag atoms outside the core, substantiating the formation of an inner shell closer to AgIn_*y*_Ga_1-*y*_S_2_ or AgGaS_2_ (Fig. 5c,f). Similar to those of the AgIn_*x*_Ga_1-*x*_S_2_/AgGaS_2_ QDs, the shell regions of the AgIn_*x*_Ga_1-*x*_S_2_/AgGaS_2_/ZnS QDs also exhibit trace amounts of In atoms, likely due to diffusion during synthesis. Zn atoms show the highest concentrations in the outer shell surrounding the AgIn_*x*_Ga_1-*x*_S_2_/AgGaS_2_ core/inner shell. Interestingly, the compositional distributions of Ag, In, and Ga are gradual rather than discrete across the layers. From a structural perspective, this compositional gradient is expected to offer advantages such as minimizing lattice mismatch between the core and inner shell and effectively reducing the likelihood of internal defect formation. Such structural features—often referred to as compositional gradient shells or gradient alloy shells—have been widely reported in InP- and CdSe-based QDs to contribute to enhancing both the QY and stability^54–58^. As observed in the reconstructed maps and proxigram, Ga atoms are also present not only in the AgIn_*x*_Ga_1-*x*_S_2_ core and AgGaS_2_ inner shell, but also in the ZnS outer shell, thus forming a Zn_1–3/2*x*_Ga_*x*_S shell (Fig. 5). The Ga content is highest in the AgGaS_2_ inner shell and lower than the Zn content in the outer shell. Because APT inherently probes only a limited number of individual QDs and may therefore suffer from limited statistical representativeness, the compositional features identified by APT were further examined using complementary techniques. XPS analyses performed on the same samples reveal consistently higher Ga-to-Ag ratios after the Zn precursor step, evaluated from multiple core-level regions, including Ag *MNN* Auger transition (where *MNN* denotes an Auger transition involving the *M* and *N* shells) vs. Ga 2*p* and Ag 4*d* vs. Ga 3*d*. This systematic increase in the Ga-to-Ag ratios supports the formation of Ga-containing outer shells and is consistent with the Zn_1–3/2*x*_Ga_*x*_S shell formation revealed by APT (Supplementary Fig. 15 and Supplementary Table 2). Inductively coupled plasma analyses performed on the same samples show overall elemental compositions consistent with the APT results (Supplementary Tables 3 and 4). Taken together, these results indicate that the structural and compositional features identified by APT are not limited to a small number of individual QDs but are representative of overall sample.

Interestingly, directly comparing two individual QDs reveals compositional differences, even among co-synthesized QDs. These differences are particularly pronounced in the core region, with noticeable variations in the Ag, In, and Ga contents; some QDs exhibit cores with a very low Ga content (Figs. 4d and 5c). Similar results were observed in the AgIn_*x*_Ga_1-*x*_S_2_/AgGaS_2_ QDs, albeit to a lesser extent. The pronounced inhomogeneity in the cores likely originates from the intrinsic complexity of the quaternary Ag–In–Ga–S system. The synthesis of the core simultaneously involves multiple cation precursors with different reactivities, and the cores are formed under a continuous heating process. Previous studies have also shown that AgIn_*x*_Ga_1-*x*_S_2_ core formation proceeds through intermediate phases and cation exchange processes rather than a simple single-step growth process^18,53,59^. More specifically, AgGaS_2_ or AgInS_2_ seeds form initially, followed by the incorporation of In and Ga to generate the chalcopyrite AgIn_*x*_Ga_1-*x*_S_2_ lattice, which is likely to promote local compositional fluctuations within the core. Furthermore, the subsequent ZnS precursor step introduces an additional thermal treatment, which can promote cation diffusion and redistribution, thereby amplifying pre-existing compositional fluctuations within the cores.

Consistent with the APT observations, STEM–EDS line profiles collected from multiple QDs reveal noticeable particle-to-particle variations in the relative Ag, In, and Ga distributions (Supplementary Figs. 16 and 17). For each QD, several independent line profiles were extracted and compared, all of which show dot-to-dot compositional fluctuations. These results indicate that the observed compositional inhomogeneity is an intrinsic feature of the synthesized quaternary QDs, thereby supporting the features revealed by APT. A deviation from the ideal stoichiometry in the core can change the emission wavelengths. Thus, such compositional inhomogeneity in the quaternary semiconductor cores may contribute to the inhomogeneous spectral broadening of the UV–Vis absorption and PL bands for both QDs, along with other factors such as the size distribution. Our APT analysis directly shows compositional inhomogeneity among the cores and highlights the importance of controlling such variations, along with defects, to improve the color purity of the green emission.

Our analytical findings have thus far proved that the outer shell in AgIn_*x*_Ga_1-*x*_S_2_/AgGaS_2_/ZnS QDs is indeed a Zn_1–3/2*x*_Ga_*x*_S shell rather than a ZnS shell. The unintended formation of this Zn_1–3/2*x*_Ga_*x*_S shell can be ascribed to cation exchange between Zn^2+^ and both Ag^+^ and Ga^3+^, with Zn^2+^ ions partially replacing both Ag^+^ and Ga^3+^ ions in the AgGaS_2_ shell during the ZnS precursor step (Supplementary Fig. 18). During the initial stages of this process, Zn^2+^ ions approach the AgGaS_2_ surface and replace Ag^+^ and Ga^3+^ ions, which occupy tetrahedral coordination sites in the AgGaS_2_ lattice. These two ions are progressively substituted by Zn^2+^ ions while maintaining charge neutrality, leading to a gradual transition in the composition of the outer shell into Zn_1–3/2*x*_Ga_*x*_S. This process forms a compositionally mixed Zn_1–3/2*x*_Ga_*x*_S outer shell with a chalcopyrite structure, which is the overall crystallographic framework of the AgGaS_2_ inner shell^18,53,60–65^. Fortuitously, the formation of Zn_1–3/2*x*_Ga_*x*_S with a chalcopyrite-like structure, which is the same as that of AgGaS_2_, is crystallographically preferable over zincblende-structured ZnS. This structural compatibility between Zn_1–3/2*x*_Ga_*x*_S and AgGaS_2_ is expected to minimize lattice mismatch, thus reducing lattice distortion and residual strain. Furthermore, this explains why the STEM–HAADF analysis revealed that the overall size of the QDs remained nearly unchanged after the ZnS precursor step, while their crystallinity was preserved throughout the QDs, thus verifying the formation of the Zn_1–3/2*x*_Ga_*x*_S shell, which is structurally similar to ZnGaS_2_, via cation exchange. The emergence of the Zn_1–3/2*x*_Ga_*x*_S outer shell via cation exchange offers a plausible origin for the long-wavelength emission tail observed after the ZnS precursor step. In our system, the substitution of Ag^+^ and Ga^3+^ by Zn^2+^ during shell formation inherently introduces cation vacancies and local charge-compensating defects. Consistent with our APT observations, the inward diffusion of Zn^2+^ into the interior of the QDs further induces cation exchange and promotes the formation of such defects. When these defects form in the vicinity of In-rich regions, where carriers are preferentially confined, they can introduce intragap trap states that locally perturb the confinement potential and promote trap-assisted recombination. These defect-related states are commonly associated with donor–acceptor-pair-like recombination pathways that give rise to long-wavelength emission in I–III–VI_2_ semiconductors^23,38–40,66–68^.

In terms of the band gap energy, the Zn_1–3/2*x*_Ga_*x*_S shell typically exhibits a slightly lower band gap than ZnS ( ≈ 3.5 eV), even at the nanoscale^62,65,69–72^. The formation of the compositionally mixed Zn_1–3/2*x*_Ga_*x*_S layer is therefore expected to adequately preserve the quantum efficiency, with negligible weakening of charge confinement, consistent with Type-I-like band alignment. Moreover, the spontaneous formation of the Zn_1–3/2*x*_Ga_*x*_S shell could significantly reduce crystallographic or interfacial defects that might arise from the structural differences between the inner and outer shells. By creating a more seamless interface with the AgGaS_2_ inner shell, the Zn_1–3/2*x*_Ga_*x*_S shell may enhance the stability of the QDs under ambient conditions and contribute to a more stable and efficient green luminescence. This optical stability is particularly advantageous for optoelectronic applications where long-term emission reliability is critical.

### Trace detection of residues in AgIn_*x*_Ga_1-*x*_S_2_/AgGaS_2_/ZnS QDs

**Fig. 6:**
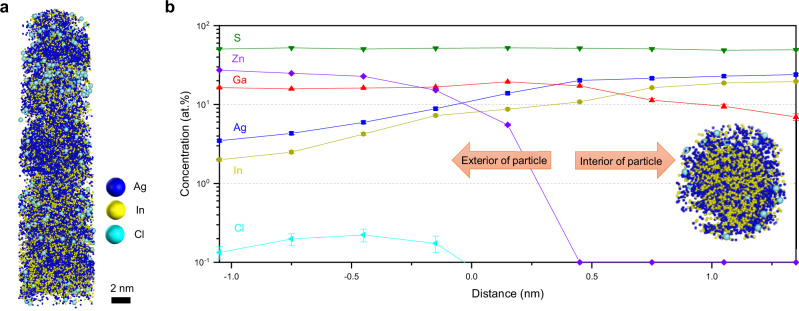
Cl residues in AgIn_*x*_Ga_1-*x*_S_2_/AgGaS_2_/ZnS QDs. **a** Cl atoms distribution in 3D atom map and **b** proxigram obtained from Ag iso-surface. Cl atoms, originating from the ZnCl_2_ precursor, are detected in trace amounts, primarily in the outermost part of the Zn_1–3/2*x*_Ga_*x*_S shell. Inset: Cross-sectional 3D atom map. Colors: Ag, blue; In, yellow; Ga, red; S, green; Zn, violet; Cl, cyan. Scale bars: 2 nm in (**a**). Error bars in (**b**) indicate one-sigma counting statistics based on the detected ion counts within each sampling bin. The analysis is based on a single dataset without independent replicate measurements. Source data are provided as a Source Data file.

To investigate the possibility of trace elements remaining in the nanoscale QDs, we evaluated the distribution of Cl atoms. As shown in Fig. 6 and Supplementary Fig. 19, Cl atoms are detected only in the AgIn_*x*_Ga_1-*x*_S_2_/AgGaS_2_/ZnS QDs and are localized in the outer shell region in only trace amounts. The Cl atoms, derived from the ZnCl_2_ precursor used in the ZnS precursor step, show a strong spatial correlation with Zn atoms in the APT analysis, whereas no comparable correlation is observed with Ag, In, or Ga atoms. XPS measurement further confirms that Cl is absent in AgIn_*x*_Ga_1-*x*_S_2_ cores and nearly absent in AgIn_*x*_Ga_1-*x*_S_2_/AgGaS_2_ QDs, whereas the Cl signal is pronounced in the AgIn_*x*_Ga_1-*x*_S_2_/AgGaS_2_/ZnS QDs, exhibiting metal–Cl chemical states attributable to Zn–Cl-related species (Supplementary Fig. 20 and Supplementary Table 2). These Cl species can be both detrimental and beneficial to the AgIn_*x*_Ga_1-*x*_S_2_/AgGaS_2_/ZnS QDs. As for the detrimental effects, residual Cl may introduce lattice defects and surface trap states, which can serve as non-radiative recombination pathways, ultimately reducing the QY and optical stability of the QDs^73,74^. Conversely, in the absence of such detrimental effects, Cl can play a beneficial role by acting as a surface passivation agent or inorganic ligand^75–77^. Given these opposing effects, the incorporation of residual elements such as Cl must be carefully controlled by monitoring both their amounts and spatial distribution. Achieving an optimal balance in the incorporation of residues can maximize their passivating and stabilizing effects while minimizing potential adverse impacts on QD performance.

Although our study only demonstrated the 3D distribution of trace elements remaining after synthesis, APT is expected to be readily applicable to a broad range of nanostructures, particularly those with complex heterostructures. Such applications will enable detailed elucidation of the spatial distribution, segregation behavior, and interfacial incorporation of trace elements or dopants, thereby providing critical insights and design guidelines for the structural and electronic optimization of advanced nanomaterials.

In this study, we elucidated the internal structure and 3D elemental distribution of AgIn_*x*_Ga_1-*x*_S_2_-based QDs, which have a heterostructure in core/shell geometry, via APT analysis. Both AgIn_*x*_Ga_1-*x*_S_2_/AgGaS_2_ and AgIn_*x*_Ga_1-*x*_S_2_/AgGaS_2_/ZnS QDs exhibited compositional gradients across layers and interfaces rather than discrete boundaries. Such gradient interfaces minimize lattice mismatch between adjacent layers, thereby reducing potential internal defects and enabling the realization of highly luminescent AgIn_*x*_Ga_1-*x*_S_2_-based QDs. For AgIn_*x*_Ga_1-*x*_S_2_/AgGaS_2_ core/shell structured QDs, we identified an Ag-deficient shell on the surface of the AgGaS_2_ shell, which could be attributed to the differences in the precursor reactivity during synthesis. For the AgIn_*x*_Ga_1-*x*_S_2_/AgGaS_2_/ZnS core/shell/shell structured QDs, we revealed the presence of compositional inhomogeneity among AgIn_*x*_Ga_1-*x*_S_2_ cores. Additionally, a Zn_1–3/2*x*_Ga_*x*_S outer shell was found to form spontaneously through cation exchange during the ZnS precursor step for outer shell formation, and this shell effectively confined charge carriers within the core with its Type-I band alignment. These findings provide insights into the atomic-scale incorporation and diffusion behavior in QDs, offering guidance for optimizing the design and performance for advanced optoelectronic applications.

## Methods

### Materials

Gallium(III) acetylacetonate (Ga(acac)_3_, 99.99%, Sigma-Aldrich), gallium(III) chloride (GaCl_3_, 98%, Allegra), silver acetate (AgOAc, 99.99% trace metal basis, Sigma-Aldrich), 1-octadecene (ODE, 90%, Sigma-Aldrich), sulfur powder (99.98%, Sigma-Aldrich), n-trioctylphosphine (TOP, 90%, Strem), oleylamine (OAm, ≥98%, Allgre), toluene (anhydrous, 99.8%, Sigma-Aldrich), *N*,*N*’-dimethylthiourea (DMTU, 97% Tokche), indium acetate (In(OAc)_3_, 99.99%, Sigma-Aldrich), and ethanol (99.5%, Samchun Chemical) were used as purchased.

### Precursor preparation

A 0.06 M Ag/OAm stock solution was prepared by dissolving AgOAc (6 mmol) in 100 mL of OAm, and the mixture was evacuated at 50 °C. In addition, a 1 M S/OAm stock solution was prepared by dissolving S powder (0.1 mol) in 100 mL of OAm under vacuum. Finally, 1 M GaCl_3_/TOP and 0.5 M ZnCl_2_/TOP stock solutions were prepared by separately dissolving GaCl_3_ (0.1 mol) and ZnCl_2_ (0.05 mol) powders in 100 mL of TOP, respectively.

### Synthesis of the AgIn_x_Ga_1-x_S cores and AgIn_*x*_Ga_1-*x*_S_2_/AgGaS_2_ QDs

AgIn_*x*_Ga_1-*x*_S_2_-core QDs were synthesized by injecting a S precursor into a mixture of Ag, In, and Ga precursors, following a procedure similar to methods reported in refs. ^18–20^. First, AgOAc, In(OAc)_3_, and Ga(acac)_3_ were mixed in 100 mL of OAm in a reaction flask, and the mixture was evacuated at room temperature for 10 min. The flask was then filled with N_2_ gas, and 40 mL of the 1 M S/OAm stock solution was swiftly injected into the flask. Next, the flask was heated to 240 °C, and the temperature was maintained until the desired particle size was achieved, while monitoring the reaction progress using UV–Vis absorption and PL spectrometry. The resulting AgIn_x_Ga_1-x_S cores were centrifuged with acetone and re-dispersed in toluene. The molar ratio of Ga to In precursors was adjusted between 1.0 and 1.4 to tune the PL peak wavelength to ≈530 nm.

Subsequently, to coat the cores with the AgGaS_2_ shell, a flask containing 2.3 × *g* of DMTU and 200 mL of an OAm/ODE (1:1) solvent mixture was evacuated at 120 °C for 10 min. Then, it was filled with N_2_ gas and heated to 260 °C. When it reached this temperature, the 1 M GaCl_3_/TOP solution, the 0.06 M Ag/OAm solution, and the AgIn_x_Ga_1-x_S core QDs in toluene ( ≈ 5 mL) were injected into the flask, and the temperature was maintained for 100 min. The final AgIn_*x*_Ga_1-*x*_S_2_/AgGaS_2_ QDs were centrifuged at 3760 × *g* with the addition of excess ethanol and re-dispersed in toluene for subsequent coating with ZnS. The Ag/Ga and Ga/S precursor ratios were 0.04 and 0.8, respectively.

### Synthesis of the AgIn_*x*_Ga_1-*x*_S_2_/AgGaS_2_/ZnS QDs

First, 0.25 × *g* of DMTU and 80 mL of OAm were loaded into a reaction flask, and the mixture was evacuated at 120 °C and heated to 210 °C under N_2_ flow. Then, the 0.5 M ZnCl_2_/TOP solution and the AgIn_*x*_Ga_1-*x*_S_2_/AgGaS_2_ core/shell QDs in toluene ( ≈ 10 mL) were quickly injected into the flask, and the temperature was maintained for 100 min. The Zn and S precursors were injected in equal molar amounts.

### Optical property characterization

UV–Vis and PL measurements were conducted with a UV-2600 (Shimadzu) and F-7100 (HITACHI), respectively. The absolute PL QYs of the QDs dispersed in toluene and QD–acrylate composite films were measured using a quantum efficiency measurement system (Otsuka, QE-2100) equipped with an integrating half sphere with excitation at 450 nm.

### STEM observations

Colloidal AgIn_*x*_Ga_1-*x*_S_2_/AgGaS_2_ and AgIn_*x*_Ga_1-*x*_S_2_/AgGaS_2_/ZnS QDs were dispersed in hexane and deposited on TEM grids with a carbon support film (Tedpella, P/N 01824). Spherical aberration (Cs)-corrected STEM (FEI-Titan Cubed) was performed in the HAADF mode at 300 kV. High-resolution STEM images were acquired using an 80 pA current probe. STEM–EDS elemental maps were obtained using a 130 pA current probe over a total acquisition time of 10 min. The average particle size was determined by measuring the diameters of more than 300 randomly selected QDs from HAADF–STEM images using ImageJ 1.53 software.

### APT specimen preparation

Needle-shaped specimens for APT analysis were prepared from dried QDs in powder form using the focused ion beam (FIB) lift-out method (Helios5 HX, Thermo Fisher Scientific). Initially, a 100 nm-thick Pt layer was deposited using an electron beam, followed by depositing a 1 μm-thick Pt layer with a Ga-ion beam to passivate a region of interest measuring 12 × 1.7 μm on the sample surface. The target region was then extracted and mounted onto a sharpened W tip. The specimens on the W tip were further shaped into a needle-like geometry using an annular milling pattern (30 kV, 80 pA). To refine the samples and reduce damage induced by the Ga-ion beam, the region of interest was thinned again with an annular pattern at 5 kV and 8 pA. The morphology and dispersion of the QDs prepared by this procedure were confirmed by HAADF–STEM (Supplementary Fig. 21).

### APT analysis

APT analysis was conducted using a CAMECA atom probe (Invizo 6000) operating in the deep-UV laser mode (*λ* = 257.5 nm) with a pulse repetition rate of 200 kHz and a base temperature of 50 K. A laser pulse energy of 100 pJ was applied, achieving a detection rate of 0.01 atoms pulse^−1^. The acquired data were reconstructed and analyzed using CAMECA AP Suite 6.3 software. All elemental species constituting the AgIn_*x*_Ga_1-*x*_S_2_-based QDs, as well as C-related ions, were identified from the mass spectra and assigned before reconstruction. The reconstructions were performed using a shank-angle-based protocol. SEM and HAADF–STEM images were utilized to guide the reconstruction and constrain the specimen geometry, thereby enhancing the accuracy of the APT reconstruction process. No artificial geometric constraints were imposed (e.g., forcing spherical particle shapes) during the reconstruction process. The slightly ellipsoidal morphologies observed in the APT datasets were therefore preserved as reconstructed rather than being artificially forced into an idealized geometry and are attributed to known trajectory aberration and local magnification effects.

### Reporting summary

Further information on research design is available in the Nature Portfolio Reporting Summary linked to this article.

## Supplementary information

Supplementary Information

Reporting Summary

Transparent Peer Review file

## Source data

Source Data

## Data Availability

The data that support the findings of this study are available from the corresponding authors upon request. Source data are provided with this paper.

## References

[1] Dai, X., Deng, Y., Peng, X. & Jin, Y. Quantum-dot light-emitting diodes for large-area displays: towards the dawn of commercialization. *Adv. Mater.* **29**, 1607022 (2017). 10.1002/adma.201607022 28256780

[2] Rosenthal, S. J., Chang, J. C., Kovtun, O., McBride, J. R. & Tomlinson, I. D. Biocompatible quantum dots for biological applications. *Chem. Biol.* **18**, 10–24 (2011). 21276935 10.1016/j.chembiol.2010.11.013 PMC3752999

[3] Jamieson, T. et al. Biological applications of quantum dots. *Biomaterials* **28**, 4717–4732 (2007). 17686516 10.1016/j.biomaterials.2007.07.014

[4] Kagan, C. R., Lifshitz, E., Sargent, E. H. & Talapin, D. V. Building devices from colloidal quantum dots. *Science* **353**, aac5523 (2016). 27563099 10.1126/science.aac5523

[5] Ning, Z. et al. Quantum-dot-in-perovskite solids. *Nature* **523**, 324–328 (2015). 26178963 10.1038/nature14563

[6] Vasudevan, D., Gaddam, R. R., Trinchi, A. & Cole, I. Core–shell quantum dots: properties and applications. *J. Alloys Compd.* **636**, 395–404 (2015).

[7] Tessier, M. D., Dupont, D., De Nolf, K., De Roo, J. & Hens, Z. Economic and size-tunable synthesis of InP/ZnE (E = S, Se) colloidal quantum dots. *Chem. Mater.* **27**, 4893–4898 (2015).

[8] Kovalenko, M. V. et al. Prospects of nanoscience with nanocrystals. *ACS Nano* **9**, 1012–1057 (2015). 25608730 10.1021/nn506223h

[9] Yang, Z. et al. Recent advances in quantum dot-based light-emitting devices: challenges and possible solutions. *Mater. Today* **24**, 69–93 (2019).

[10] Zhang, C. Y., Yeh, H. C., Kuroki, M. T. & Wang, T. H. Single-quantum-dot-based DNA nanosensor. *Nat. Mater.* **4**, 826–831 (2005). 16379073 10.1038/nmat1508

[11] Wang, X., Yu, J. & Chen, R. Optical characteristics of ZnS passivated CdSe/CdS quantum dots for high photostability and lasing. *Sci. Rep.* **8**, 17323 (2018). 30470827 10.1038/s41598-018-35768-8 PMC6251909

[12] Alivisatos, A. P. Semiconductor clusters, nanocrystals, and quantum dots. *Science* **271**, 933–937 (1996).

[13] Baskoutas, S. & Terzis, A. F. Size-dependent band gap of colloidal quantum dots. *J. Appl. Phys.* **99**, 013708 (2006).

[14] Peng, H., Zhang, L., Soeller, C. & Travas-Sejdic, J. Preparation of water-soluble CdTe/CdS core/shell quantum dots with enhanced photostability. *J. Lumin.* **127**, 721–726 (2007).

[15] Lim, J. et al. Influence of shell thickness on the performance of light-emitting devices based on CdSe/Zn_1-*X*_Cd_*X*_S core/shell heterostructured quantum dots. *Adv. Mater.* **26**, 8034–8040 (2014). 25381683 10.1002/adma.201403620

[16] Wegner, K. D. et al. Influence of the core/shell structure of indium phosphide based quantum dots on their photostability and cytotoxicity. *Front. Chem.* **7**, 466 (2019). 31316974 10.3389/fchem.2019.00466 PMC6610543

[17] Peng, X., Schlamp, M. C., Kadavanich, A. V. & Alivisatos, A. P. Epitaxial growth of highly luminescent CdSe/CdS core/shell nanocrystals with photostability and electronic accessibility. *J. Am. Chem. Soc.* **119**, 7019–7029 (1997).

[18] Lee, H. J. et al. Coherent heteroepitaxial growth of I-III-VI_2_ Ag(In,Ga)S_2_ colloidal nanocrystals with near-unity quantum yield for use in luminescent solar concentrators. *Nat. Commun.* **14**, 3779 (2023). 37355655 10.1038/s41467-023-39509-y PMC10290649

[19] Kameyama, T. et al. Wavelength-tunable band-edge photoluminescence of nonstoichiometric Ag–In–S nanoparticles via Ga^3+^ doping. *ACS Appl. Mater. Interfaces* **10**, 42844–42855 (2018). 30508368 10.1021/acsami.8b15222

[20] Kim, Y. et al. Green Ag–In–Ga–S quantum dots as highly absorption-capable, efficient, and color-pure emitters. *Chem. Eng. J.* **486**, 150219 (2024).

[21] Hu, Z. et al. Aqueous synthesis of 79% efficient AgInGaS/ZnS quantum dots for extremely high color rendering white light-emitting diodes. *J. Mater. Sci. Technol.* **134**, 189–196 (2023).

[22] Motomura, G. et al. Quantum-dot light-emitting diodes exhibiting narrow-spectrum green electroluminescence by using Ag–In–Ga–S/GaS_x_ quantum dots. *ACS Appl. Mater. Interfaces* **15**, 8336–8344 (2023). 36732881 10.1021/acsami.2c21232

[23] Kim, J. H. et al. Synthesis of widely emission-tunable Ag–Ga–S and its quaternary derivative quantum dots. *Chem. Eng. J.* **347**, 791–797 (2018).

[24] Hoisang, W., Uematsu, T., Torimoto, T. & Kuwabata, S. Luminescent quaternary Ag(In_*x*_Ga_1–*x*_)S_2_/GaS_*y*_ Core/shell quantum dots prepared using dithiocarbamate compounds and photoluminescence recovery via post treatment. *Inorg. Chem.* **60**, 13101–13109 (2021). 34410714 10.1021/acs.inorgchem.1c01513

[25] Weigert, F. et al. Combining HR-TEM and XPS to elucidate the core–shell structure of ultrabright CdSe/CdS semiconductor quantum dots. *Sci. Rep.* **10**, 20712 (2020). 33244030 10.1038/s41598-020-77530-z PMC7692488

[26] Abel, K. A. et al. Probing the structure of colloidal core/shell quantum dots formed by cation exchange. *J. Phys. Chem. C* **116**, 3968–3978 (2012).

[27] McBride, J., Treadway, J., Feldman, L. C., Pennycook, S. J. & Rosenthal, S. J. Structural basis for near unity quantum yield core/shell nanostructures. *Nano Lett.* **6**, 1496–1501 (2006). 16834437 10.1021/nl060993k

[28] Bals, S. et al. Three-dimensional atomic imaging of colloidal core–shell nanocrystals. *Nano Lett.* **11**, 3420–3424 (2011). 21786766 10.1021/nl201826e

[29] Cooper, J. K., Franco, A. M., Gul, S., Corrado, C. & Zhang, J. Z. Characterization of primary amine capped CdSe, ZnSe, and ZnS quantum dots by FT-IR: determination of surface bonding interaction and identification of selective desorption. *Langmuir* **27**, 8486–8493 (2011). 21631120 10.1021/la201273x

[30] Farahmandzadeh, F., Molaei, M., Alehdaghi, H., Karimipour, M. & Shamsi, A. Effect of concentration and shell thickness on the optical behavior of aqueous CdTe/ZnSe core/shell quantum dots (QDs) exposed to ionizing radiation. *Luminescence* **37**, 431–439 (2022). 34994062 10.1002/bio.4189

[31] Marquis, E. A. & Hyde, J. M. Applications of atom-probe tomography to the characterisation of solute behaviours. *Mater. Sci. Eng. R Rep.* **69**, 37–62 (2010).

[32] Devaraj, A. et al. Three-dimensional nanoscale characterisation of materials by atom probe tomography. *Int. Mater. Rev.* **63**, 68–101 (2018).

[33] Gault, B., Moody, M. P., Cairney, J. M. & Ringer, S. P. *Atom Probe Microscopy*. Vol. 160 (Springer Science & Business Media, 2012).

[34] Schmidt, J. E., Peng, L., Poplawsky, J. D. & Weckhuysen, B. M. Nanoscale chemical imaging of zeolites using atom probe tomography. *Angew. Chem. Int. Ed.* **57**, 10422–10435 (2018). 10.1002/anie.201712952 PMC6519151 29718553

[35] Hu, R., Jin, S. & Sha, G. Application of atom probe tomography in understanding high entropy alloys: 3D local chemical compositions in atomic scale analysis. *Prog. Mater. Sci.* **123**, 100854 (2022).

[36] Dai, M. et al. Tunable photoluminescence from the visible to near-infrared wavelength region of non-stoichiometric AgInS_2_ nanoparticles. *J. Mater. Chem.* **22**, 12851–12858 (2012).

[37] Chen, Y. et al. Green and facile synthesis of high-quality water-soluble Ag-In-S/ZnS core/shell quantum dots with obvious bandgap and sub-bandgap excitations. *J. Alloys Compd.* **753**, 364–370 (2018).

[38] Park, S. M. et al. Red Ag-based I–III–VI quantum dots as competitive alternatives to InP emitters. *ACS Energy Lett.* **10**, 3005–3013 (2025).

[39] Farid, A. et al. One-pot synthesis of luminescent Ag−Cu−Ga−S/ZnS quantum dots bridging the cyan gap for ultrahigh-color-rendering white-light-emitting diodes. *ACS Appl. Nano Mater* **8**, 14703–14712 (2025).

[40] Park, S. et al. Suppressing tail emission from AgIn_1−*x*_Ga_*x*_S_2_/AgGaS_2_ quantum dots by GaI_3_–assisted interface reinforcement. *ACS Nano* **19**, 26831–26842 (2025). 40676965 10.1021/acsnano.5c07418

[41] Chae, B. G. et al. Direct three-dimensional observation of core/shell-structured quantum dots with a composition-competitive gradient. *ACS Nano* **12**, 12109–12117 (2018). 30474967 10.1021/acsnano.8b05379

[42] Jang, K. et al. Three-dimensional atomic mapping of ligands on palladium nanoparticles by atom probe tomography. *Nat. Commun.* **12**, 4301 (2021). 34262042 10.1038/s41467-021-24620-9 PMC8280228

[43] Kim, S. H. et al. Characterization of Pd and Pd@ Au core-shell nanoparticles using atom probe tomography and field evaporation simulation. *J. Alloys Compd.* **831**, 154721 (2020).

[44] Tedsree, K. et al. Hydrogen production from formic acid decomposition at room temperature using a Ag–Pd core–shell nanocatalyst. *Nat. Nanotechnol.* **6**, 302–307 (2011). 21478867 10.1038/nnano.2011.42

[45] Grenier, A. et al. 3D analysis of advanced nano-devices using electron and atom probe tomography. *Ultramicroscopy* **136**, 185–192 (2014). 24189616 10.1016/j.ultramic.2013.10.001

[46] Khan, M. A., Ringer, S. P. & Zheng, R. Atom probe tomography on semiconductor devices. *Adv. Mater. Interfaces* **3**, 1500713 (2016).

[47] Hatzoglou, C., Radiguet, B. & Pareige, P. Experimental artefacts occurring during atom probe tomography analysis of oxide nanoparticles in metallic matrix: quantification and correction. *J. Nucl. Mater.* **492**, 279–291 (2017).

[48] Philippe, T., Gruber, M., Vurpillot, F. & Blavette, D. Clustering and local magnification effects in atom probe tomography: a statistical approach. *Microsc. Microanal.* **16**, 643–648 (2010). 20849680 10.1017/S1431927610000449

[49] Lawitzki, R., Stender, P. & Schmitz, G. Compensating local magnifications in atom probe tomography for accurate analysis of nano-sized precipitates. *Microsc. Microanal.* **27**, 1–12 (2021). 33722337 10.1017/S1431927621000180

[50] Beinke, D., Oberdorfer, C. & Schmitz, G. Towards an accurate volume reconstruction in atom probe tomography. *Ultramicroscopy* **165**, 34–41 (2016). 27062338 10.1016/j.ultramic.2016.03.008

[51] Takahashi, J. & Kawakami, K. Position artifacts in 3D reconstruction of plate-shaped precipitates in steels depending on the analysis direction of atom probe tomography. *Surf. Interface Anal.* **53**, 982–995 (2021).

[52] Maruyama, N., Smith, G. D. W. & Cerezo, A. Interaction of the solute niobium or molybdenum with grain boundaries in α-iron. *Mater. Sci. Eng. A* **353**, 126–132 (2003).

[53] Uematsu, T., Tepakidareekul, M., Hirano, T., Torimoto, T. & Kuwabata, S. Facile high-yield synthesis of Ag–In–Ga–S quaternary quantum dots and coating with gallium sulfide shells for narrow band-edge emission. *Chem. Mater.* **35**, 1094–1106 (2023).

[54] Lee, S. H. et al. The effects of discrete and gradient mid-shell structures on the photoluminescence of single InP quantum dots. *Nanoscale* **11**, 23251–23258 (2019). 31782468 10.1039/c9nr06847c

[55] Lim, J. et al. InP@ ZnSeS, core@ composition gradient shell quantum dots with enhanced stability. *Chem. Mater.* **23**, 4459–4463 (2011).

[56] Bae, W. K., Char, K., Hur, H. & Lee, S. Single-step synthesis of quantum dots with chemical composition gradients. *Chem. Mater.* **20**, 531–539 (2008).

[57] Duan, X. et al. InP quantum dots with a strain-engineered gradient shell for enhanced optical performance and stability. *Nano Lett.* **25**, 13539–13548 (2025). 40879067 10.1021/acs.nanolett.5c03042

[58] Yu, P. et al. Highly efficient green InP-based quantum dot light-emitting diodes regulated by inner alloyed shell component. *Light Sci. Appl.* **11**, 162 (2022). 35637219 10.1038/s41377-022-00855-z PMC9151710

[59] Xie, X. et al. Narrow-bandwidth I–III–VI semiconductor nanocrystals: synthesis, luminescence and applications in quantum-dot light-emitting diodes. *Adv. Phys. Res.* **3**, 2400071 (2024).

[60] Yan, D. et al. High photoluminescence Ag-In-Ga-S quantum dots based on ZnX_2_-treated surface passivation. *Nano Res.* **17**, 7533–7541 (2024).

[61] Jang, J. S., Borse, P. H., Lee, J. S., Choi, S. H. & Kim, H. G. Indium induced band gap tailoring in AgGa_1− *x*_In_*x*_S_2_ chalcopyrite structure for visible light photocatalysis. *J. Chem. Phys*. 10.1063/1.2900984 (2008). 10.1063/1.2900984 18433268

[62] Asadullayeva, S. G., Ismayilova, N. A., Musayev, M. A. & Abbasov, I. I. Optical and electronic properties of defect chalcopyrite ZnGa_2_S_4_. *Int. J. Mod. Phys. B* **38**, 2450007 (2024).

[63] Bai, L., Lin, Z., Wang, Z., Chen, C. & Lee, M. H. Mechanism of linear and nonlinear optical effects of chalcopyrite AgGaX_2_ (X=S, Se, and Te) crystals. *J. Chem. Phys* **120**, 8772–8778 (2004). 15267809 10.1063/1.1687338

[64] Kaga, H. & Kudo, A. Cosubstituting effects of copper(I) and gallium(III) for ZnGa_2_S_4_ with defect chalcopyrite structure on photocatalytic activity for hydrogen evolution. *J. Catal.* **310**, 31–36 (2014).

[65] Sahariya, J., Kumar, P. & Soni, A. Structural and optical investigations of ZnGa_2_X_4_ (X = S, Se) compounds for solar photovoltaic applications. *Mater. Chem. Phys.* **199**, 257–264 (2017).

[66] Shen, F. et al. Photophysics and photovoltaic properties of Zn-alloyed Ag-In-S quantum dots sensitized solar cells. *J. Alloys Compd.* **922**, 166296 (2022).

[67] Rivaux, C. et al. Continuous flow aqueous synthesis of highly luminescent AgInS_2_ and AgInS_2_/ZnS quantum dots. *J. Phys. Chem. C* **126**, 20524–20534 (2022).

[68] Kameyama, T. et al. Controlling the electronic energy structure of ZnS–AgInS_2_ solid solution nanocrystals for photoluminescence and photocatalytic hydrogen evolution. *J. Phys. Chem. C* **119**, 24740–24749 (2015).

[69] Shu, C. et al. Facile synthesis and characterization of water soluble ZnSe/ZnS quantum dots for cellar imaging. *Spectrochim. Acta A Mol. Biomol. Spectrosc.* **104**, 143–149 (2013). 23266687 10.1016/j.saa.2012.11.083

[70] Asadullayeva, S. G., Jahangirli, Z. A., Naghiyev, T. G. & Mammadov, D. A. Optical and dynamic properties of ZnGa_2_S_4_. *Phys. Status Solidi.* **258**, 2100101 (2021).

[71] Hu, T., Zhu, K., Cheng, H., Teng, Y. & Pan, Z. Core–shell energy band engineering of cyan light-emitting ternary ZnGa_2_S_4_@ZnS quantum dots toward anti-counterfeiting and bioimaging applications. *J. Mater. Chem. C* **13**, 21797–21811 (2025).

[72] Yadav, A. N. & Singh, K. Investigation of photophysical properties of ternary Zn–Ga–S quantum dots: band gap versus sub-band-gap excitations and emissions. *ACS Omega* **4**, 18327–18333 (2019). 31720534 10.1021/acsomega.9b02546 PMC6844091

[73] Greaney, M. J. et al. Controlling the trap state landscape of colloidal CdSe nanocrystals with cadmium halide ligands. *Chem. Mater.* **27**, 744–756 (2015).

[74] Dirin, D. N. et al. Lead halide perovskites and other metal halide complexes as inorganic capping ligands for colloidal nanocrystals. *J. Am. Chem. Soc.* **136**, 6550–6553 (2014). 24746226 10.1021/ja5006288 PMC4524702

[75] Bae, W. K. et al. Highly effective surface passivation of PbSe quantum dots through reaction with molecular chlorine. *J. Am. Chem. Soc.* **134**, 20160–20168 (2012). 23131125 10.1021/ja309783v

[76] Li, X. et al. Bright colloidal quantum dot light-emitting diodes enabled by efficient chlorination. *Nat. Photon.* **12**, 159–164 (2018).

[77] Niu, G. et al. Inorganic halogen ligands in quantum dots: I^−^, Br^−^, Cl^−^ and film fabrication through electrophoretic deposition. *Phys. Chem. Chem. Phys.* **15**, 19595–19600 (2013). 23958930 10.1039/c3cp52678j

