# Supplementary material for: 3D mapping of compositional gradients of core-shell structures in AgInxGa1-xS2 quantum dots by atom probe tomography
Source: Nat Commun. 2026 Apr 3;17:4759. doi: 10.1038/s41467-026-71518-5 (PMC13216564; doi:10.1038/s41467-026-71518-5)
Supplement: Supplementary file 1 — Supplementary Information [file 41467_2026_71518_MOESM1_ESM.pdf]

Supplementary Information for

3D mapping of compositional gradients of core-shell structures in $AgIn_xGa_{1-x}S_2$ quantum dots by atom probe tomography

Byeong-Gyu Chae[1,†], Mihye Lim[2,3,†], Junho Lee[2], Nayoun Won[2], Soo Kyung Kwon[2], Ara Jo[2], Dong Jin Yun[1], Sangjun Lee[1], Jwa-Min Nam[3]*, Soohwan Sul[1]*, Tae-Gon Kim[2]*

[1]Analytical Engineering Group, Samsung Advanced Institute of Technology, Samsung Electronics Co., Ltd., 130 Samsung-ro, Suwon, 16678, Republic of Korea

[2]Display Solution Platform, Samsung Advanced Institute of Technology, Samsung Electronics Co., Ltd., 130 Samsung-ro, Suwon, 16678, Republic of Korea

[3]Department of Chemistry, Seoul National University, Seoul, 08826, Republic of Korea

*e-mail: jmnam@snu.ac.kr, soohwan.sul@samsung.com, taegon2.kim@samsung.com

[†]These authors contributed equally: Byeong-Gyu Chae, Mihye Lim.

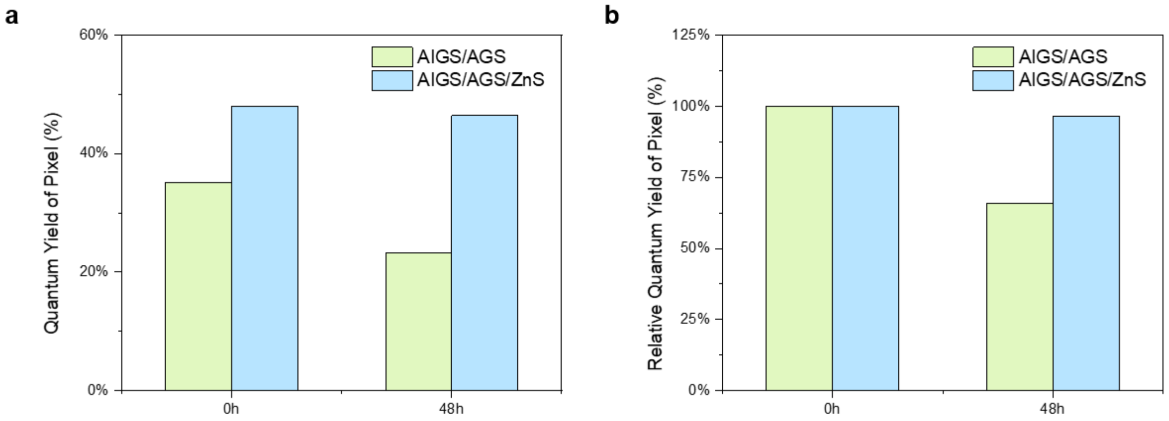

Supplementary Figure 1. Ambient-condition stability measured in the QD–acrylate composite films. a QY and **b** normalized QY after 0 and 48 h of $AgIn_xGa_{1-x}S_2/AgGaS_2$ (AIGS/AGS) and $AgIn_xGa_{1-x}S_2/AgGaS_2/ZnS$ (AIGS/AGS/ZnS) QDs.

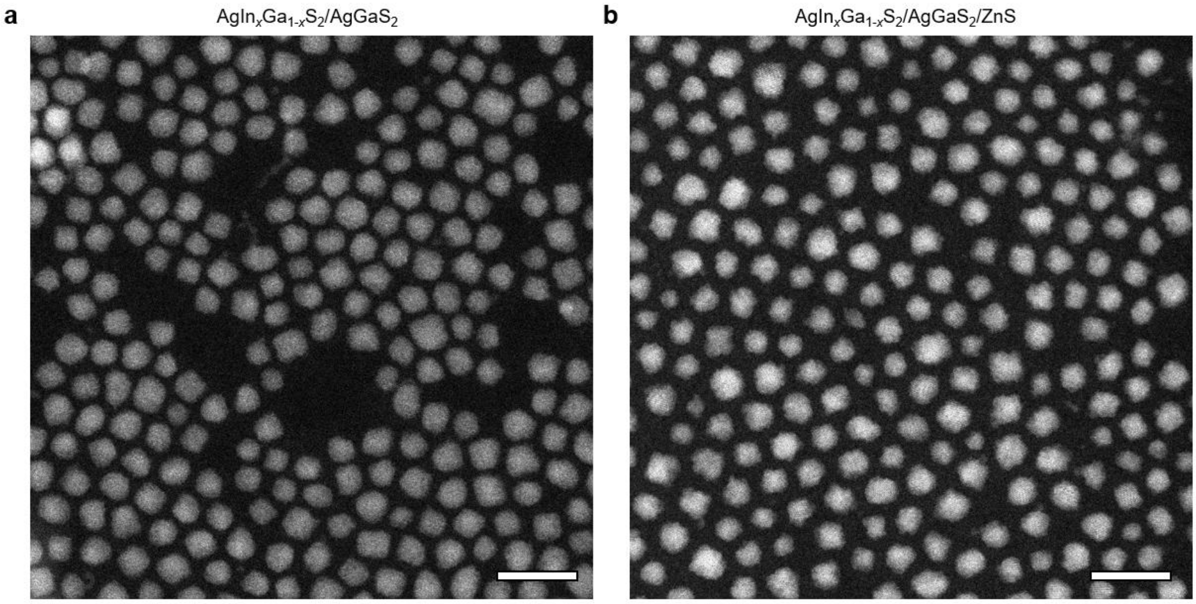

Supplementary Figure 2. HAADF–STEM image of $AgIn_xGa_{1-x}S_2/AgGaS_2$ QDs and $AgIn_xGa_{1-x}S_2/AgGaS_2/ZnS$ QDs without cropping. Low-magnification HAADF–STEM images of **a** $AgIn_xGa_{1-x}S_2/AgGaS_2$ QDs and **b** $AgIn_xGa_{1-x}S_2/AgGaS_2/ZnS$ QDs. After the ZnS precursor step, the size of the QDs changes very little. (Scale bars: 20 nm in **a, b**.)

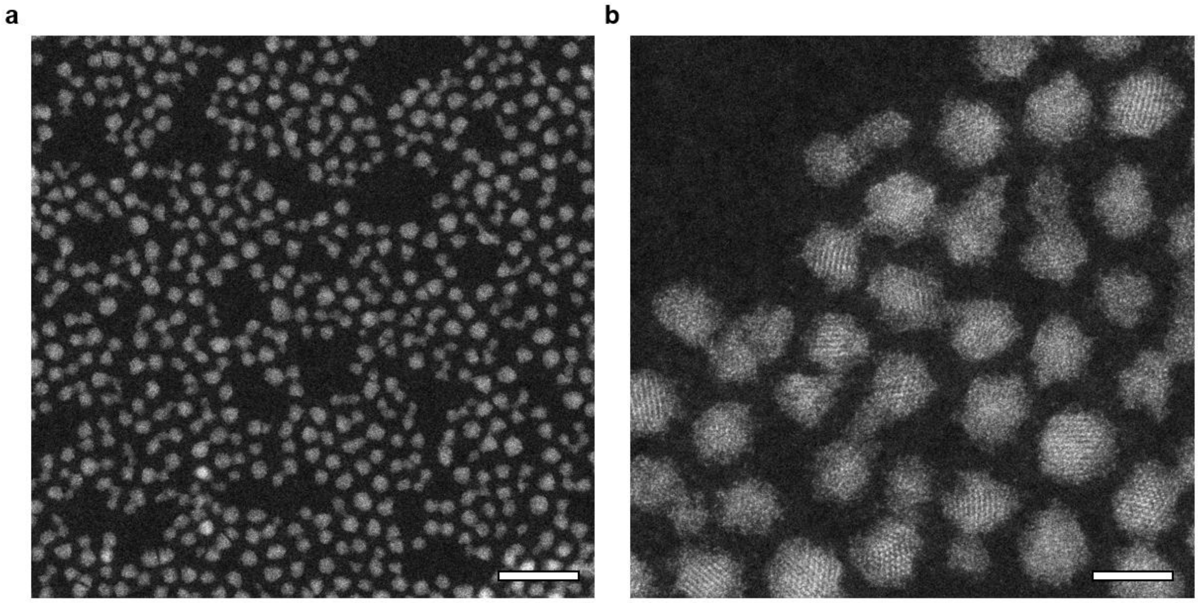

Supplementary Figure 3. HAADF–STEM image of core-only $AgIn_xGa_{1-x}S_2$ QDs. a Low-magnification and **b** high-magnification HAADF–STEM images of core-only $AgIn_xGa_{1-x}S_2$ QDs. The $AgIn_xGa_{1-x}S_2$ QDs are crystalline and exhibit an average diameter of 3.58 ± 0.59 nm. (Scale bars: 20 nm in **a** and 5nm in **b**.)

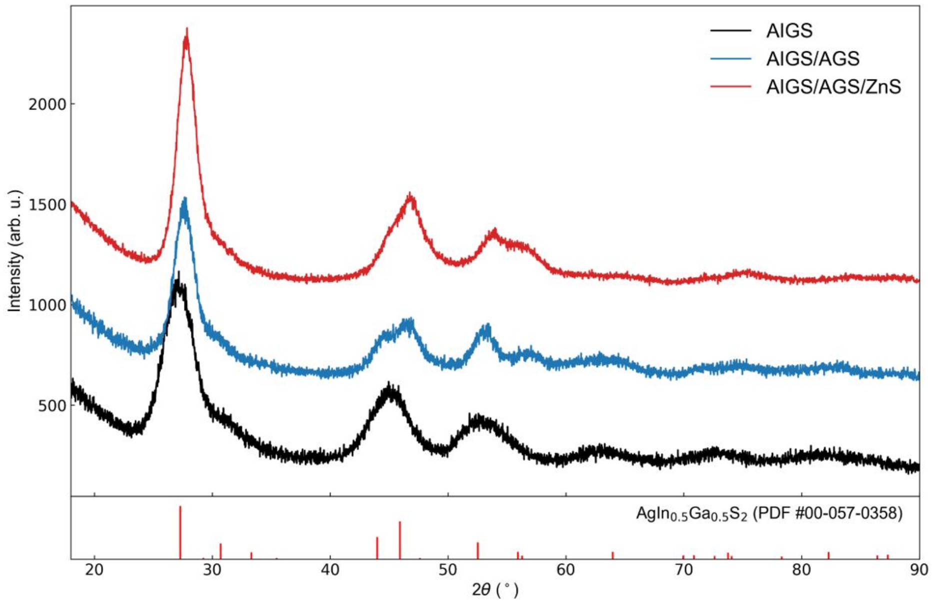

Supplementary Figure 4. XRD patterns of $AgIn_xGa_{1-x}S_2$-based QDs. XRD patterns of $AgIn_xGa_{1-x}S_2$ (AIGS), $AgIn_xGa_{1-x}S_2/AgGaS_2$ (AIGS/AGS), and $AgIn_xGa_{1-x}S_2/AgGaS_2/ZnS$ (AIGS/AGS/ZnS) QDs. All XRD patterns are consistent with a chalcopyrite-based tetragonal structure, with only minor peak shifts attributable to slight lattice parameter variations.

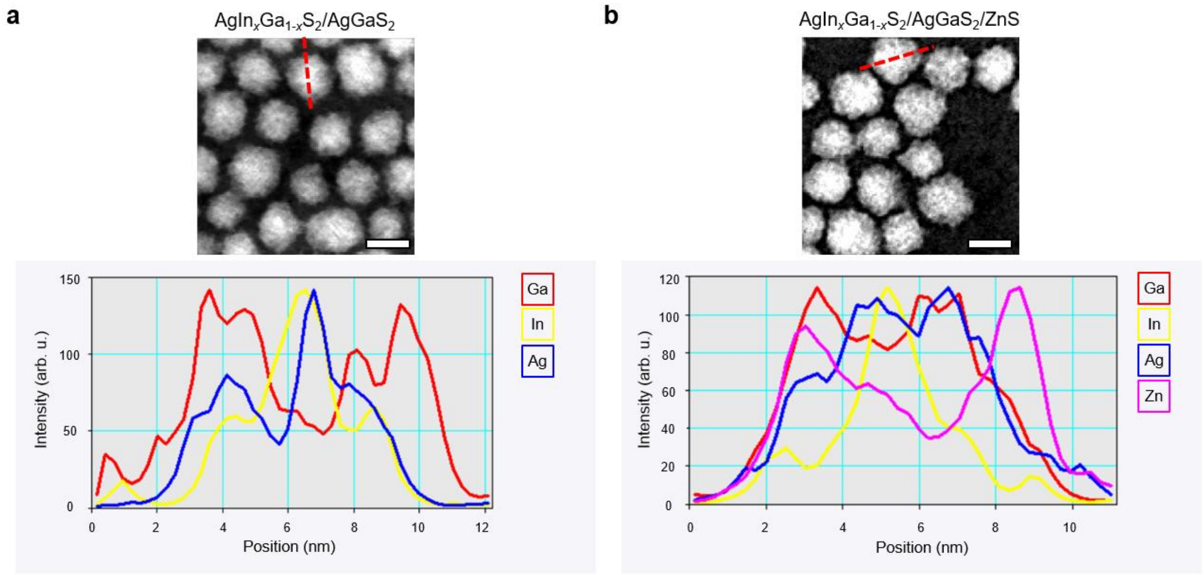

Supplementary Figure 5. STEM–EDS line profiles of AgIn$_x$Ga$_{1-x}$S$_2$/AgGaS$_2$ QDs and AgIn$_x$Ga$_{1-x}$S$_2$/AgGaS$_2$/ZnS QDs. STEM–EDS line profiles of **a** AgIn$_x$Ga$_{1-x}$S$_2$/AgGaS$_2$ QDs and **b** AgIn$_x$Ga$_{1-x}$S$_2$/AgGaS$_2$/ZnS QDs along the red dotted line in the corresponding HAADF–STEM images. (Scale bars: 7 nm in **a, b**.)

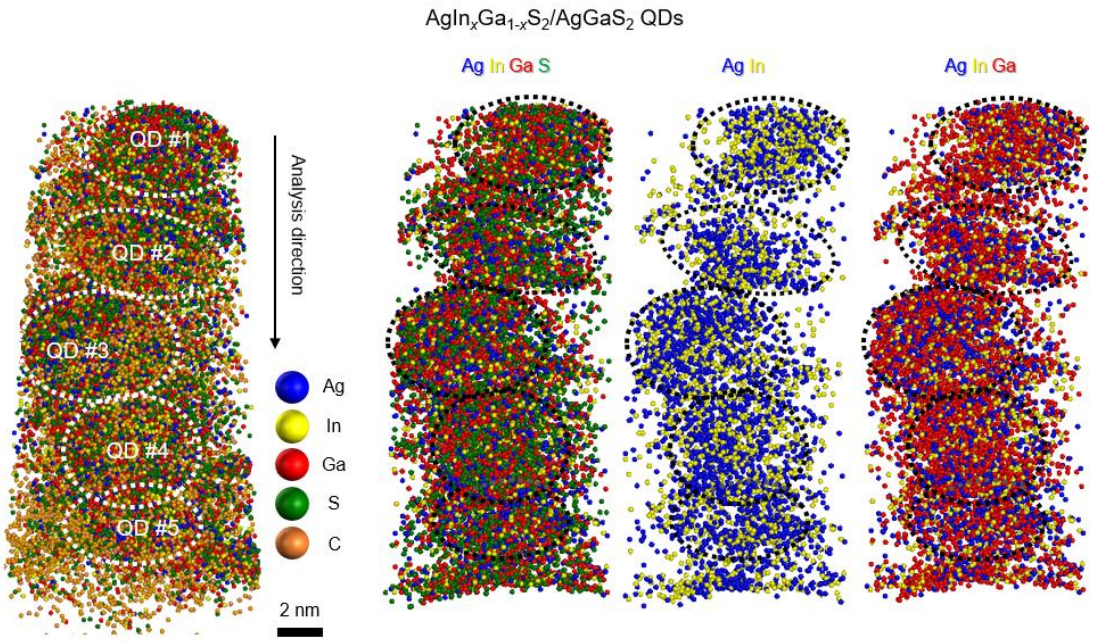

Supplementary Figure 6. APT 3D reconstructions of $AgIn_xGa_{1-x}S_2/AgGaS_2$ QDs with delineated particle boundaries. APT 3D reconstructions showing multiple $AgIn_xGa_{1-x}S_2$ /$AgGaS_2$ QDs within the analyzed volume. Dashed ovals mark the boundaries between individual QDs. In the APT specimen, the QDs are arranged in a close-packed, non-collinear configuration, which can lead to partial overlap in the projection; the similar elemental compositions of neighboring QDs further reduce the visual contrast between adjacent particles. Colors: Ag, blue; In, yellow; Ga, red; S, green; C, orange. (Scale bars: 2 nm.)

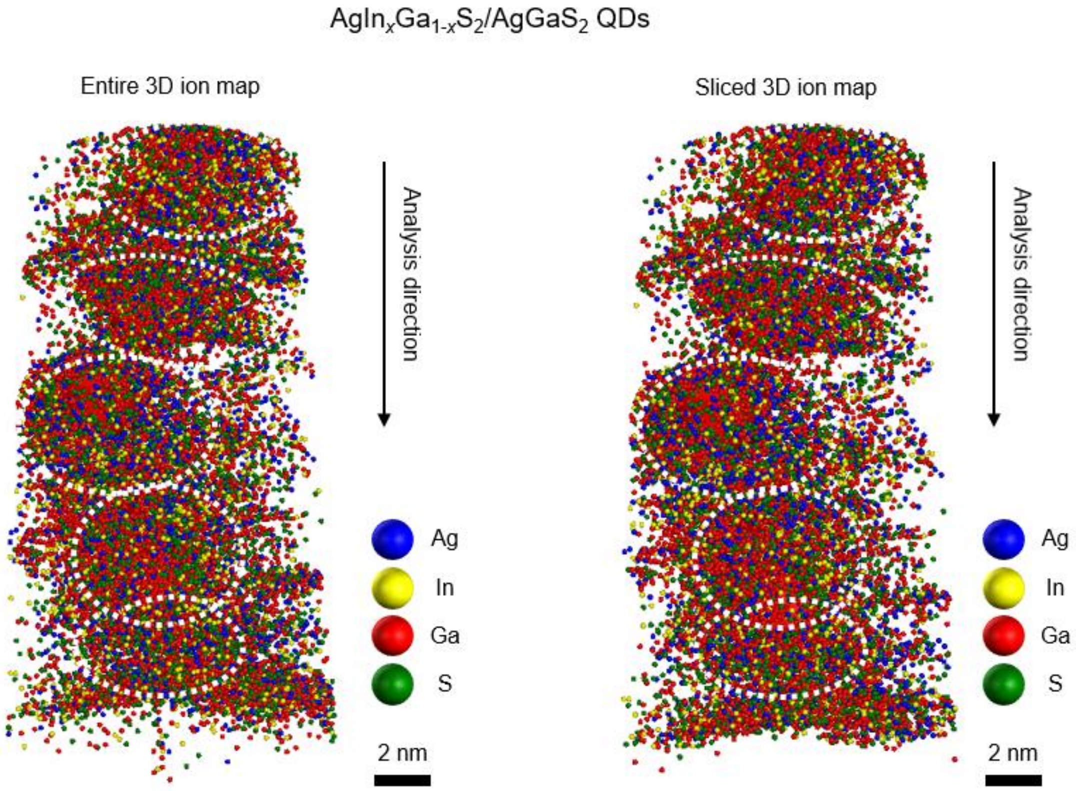

Supplementary Figure 7. APT 3D reconstructions of $AgIn_xGa_{1-x}S_2/AgGaS_2$ QDs after the removal of C- and H-related species. C- and H-related ions were excluded from the datasets for visual clarity. Both the entire reconstructed volume ($11 \times 11 \times 22$ nm^3) and a sliced 3D ion map ($6 \times 11 \times 22$ nm^3) are shown, with dashed guides indicating the approximate boundaries of individual QDs. Because the QDs are arranged in a close-packed, non-collinear configuration within the APT specimen, some particles may appear partially overlapped in the projection views. Colors: Ag, blue; In, yellow; Ga, red; S, green. (Scale bars: 2 nm.)

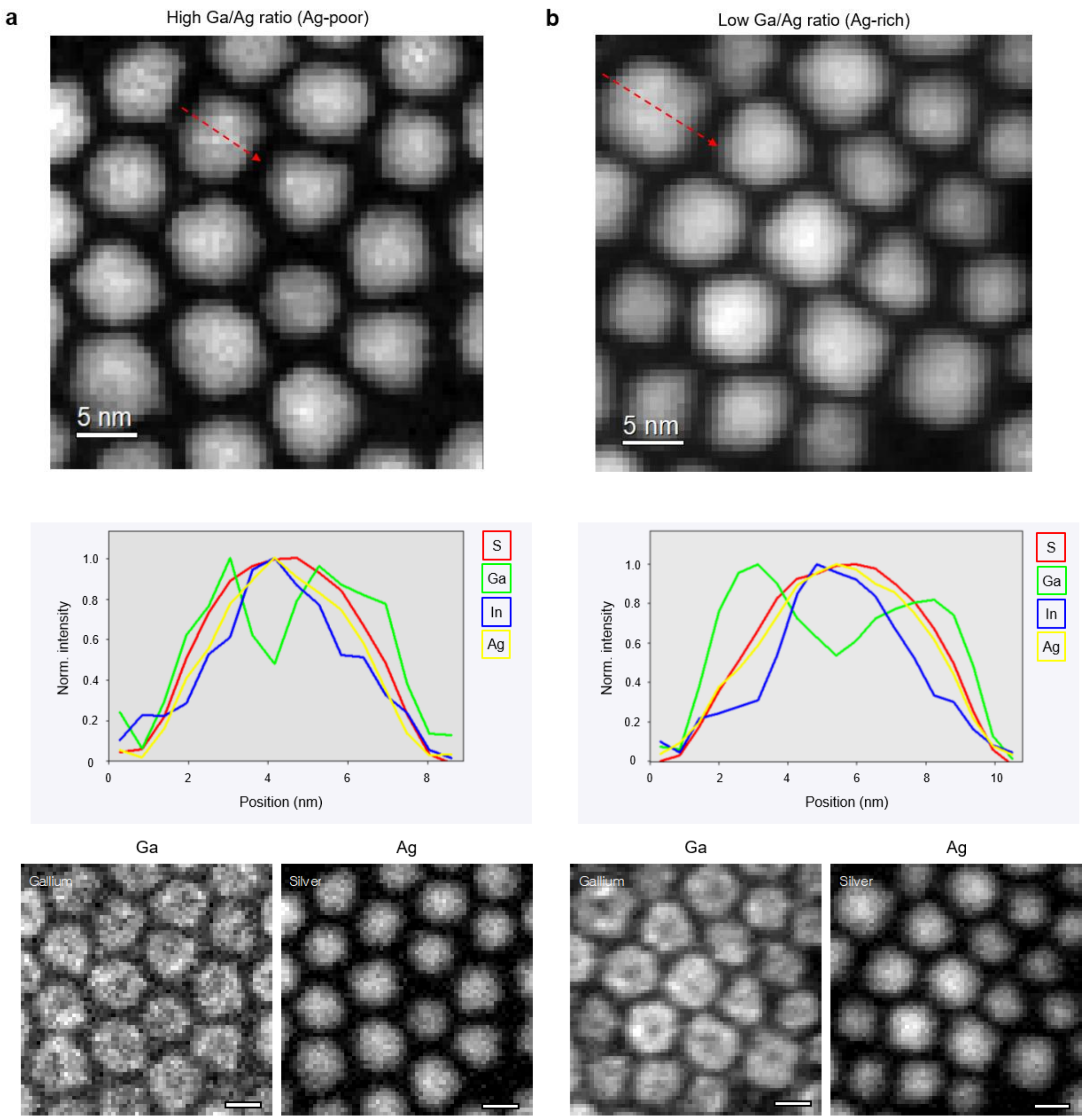

Supplementary Figure 8. STEM–EELS analysis of $AgIn_xGa_{1-x}S_2/AgGaS_2$ QDs as a function of the Ga/Ag precursor ratio. (a) Representative STEM–EELS results for high-Ga/Ag-ratio (Ag-poor) $AgIn_xGa_{1-x}S_2/AgGaS_2$ QDs and low-Ga/Ag-ratio (Ag-rich) $AgIn_xGa_{1-x}S_2/AgGaS_2$ QDs. As the Ag precursor amount increases, the overall diameter of the QDs increases, particularly because of the increasing thickness of the $AgGaS_2$ shell. Owing to the relatively low reactivity of the Ga precursor, increasing the Ag precursor under Ga-rich

conditions mainly promotes the growth of the $AgGaS_2$ shell, accompanied by increased Ag incorporation. (Scale bars: 5 nm.)

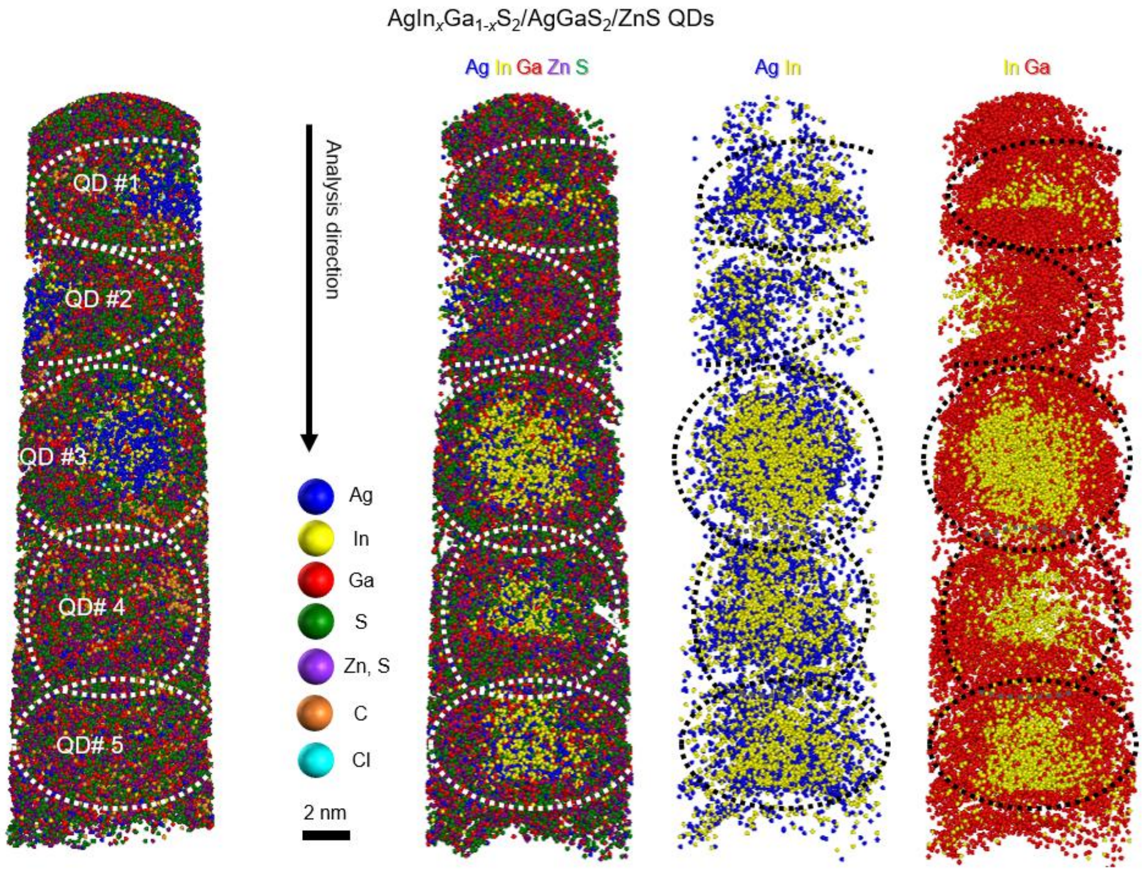

Supplementary Figure 9. APT 3D reconstructions of AgIn$_x$Ga$_{1-x}$S$_2$/AgGaS$_2$/ZnS QDs with delineated particle boundaries. APT 3D reconstructions showing multiple AgIn$_x$Ga$_{1-x}$S$_2$ /AgGaS$_2$/ZnS QDs within the analyzed volume. Dashed guides mark the boundaries between individual QDs. In the APT specimen, the QDs are arranged in a close-packed, non-collinear configuration, leading to partial overlap in the projection. Colors: Ag, blue; In, yellow; Ga, red; S, green; Zn, violet; C, orange; Cl, cyan. (Scale bars: 2 nm.)

a

b

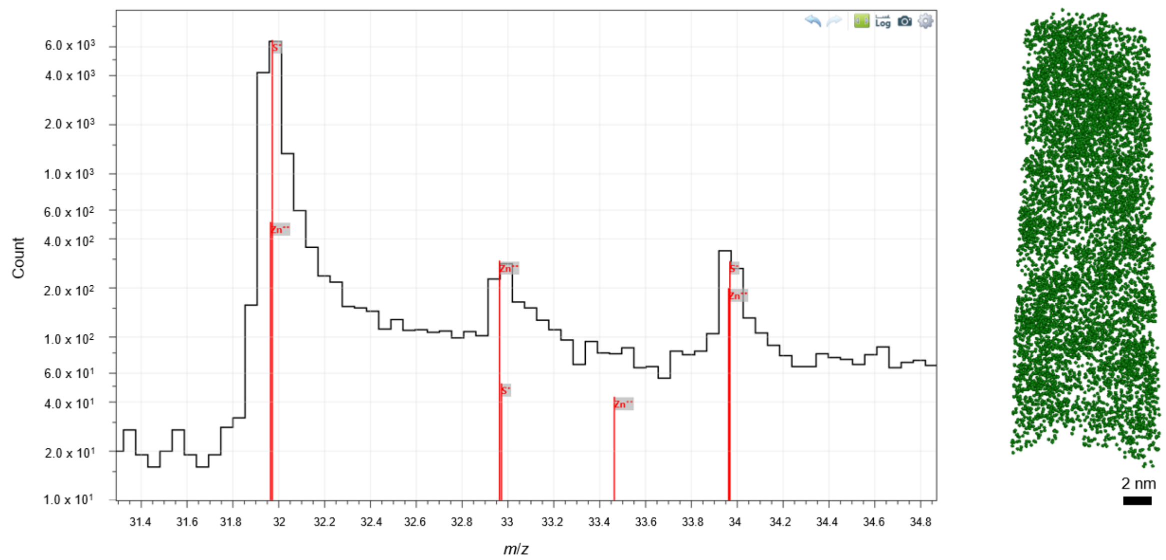

Supplementary Figure 10. APT results of S^+ in AgIn$_x$Ga$_{1-x}$S$_2$/AgGaS$_2$/ZnS QDs. a APT mass spectrum at ≈32 Da in AgIn$_x$Ga$_{1-x}$S$_2$/AgGaS$_2$/ZnS QDs. The signal of S^+ is much larger than that of Zn^{2+} at ≈32 Da. **b** 3D reconstructed map of S^+ is ≈32 Da (green, 2 nm slice-view image). S^+ ions are observed throughout the QDs, from the core to the shell. Colors: S, green. (Scale bar: 2 nm in **b**.)

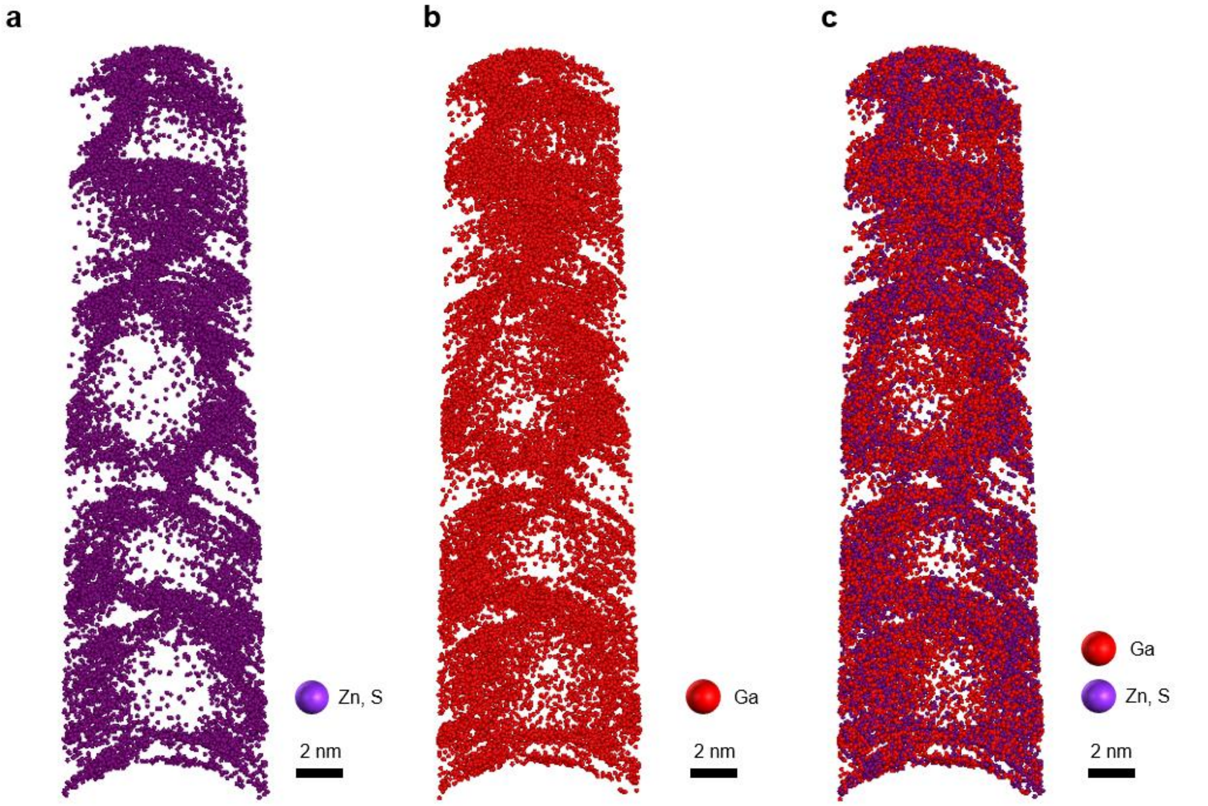

Supplementary Figure 11. Sliced 3D atom maps of $AgIn_xGa_{1-x}S_2$/$AgGaS_2$/ZnS QDs. 2 nm-thick slices of the 3D atom map of **a** Zn or S (violet, mainly Zn, at ≈64 Da), **b** Ga (red), and **c** Zn and Ga. Ga shows a broader distribution than expected. Ga is also present in significant amounts in the ZnS outer shell, again showing a broader distribution than expected. Colors: Ga, red; Zn, violet. (Scale bars: 2 nm in **a-c**.)

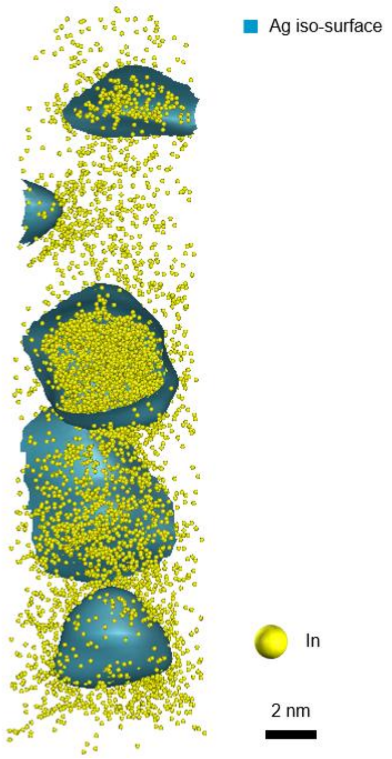

Supplementary Figure 12. 3D maps of In in $AgIn_xGa_{1-x}S_2/AgGaS_2/ZnS$ QDs. Maps of In with the Ag iso-surface, showing the distribution of In in the QDs. Colors: In, yellow. (Scale bar: 2 nm.)

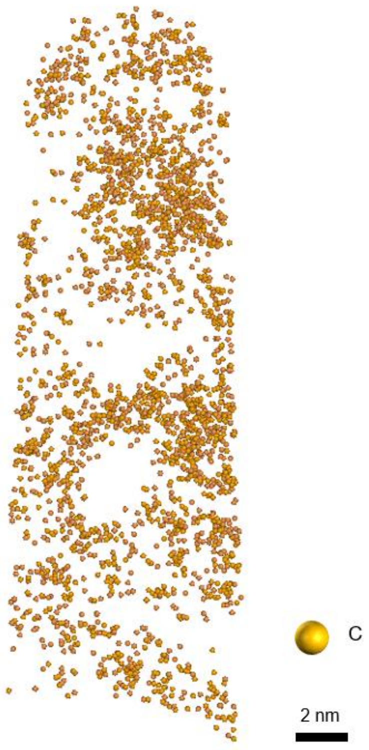

Supplementary Figure 13. Sliced 3D map of C in $AgIn_xGa_{1-x}S_2/AgGaS_2/ZnS$ QDs. 2 nm-thick slice of the 3D C map (orange), showing the distribution of C outside the QDs. Colors: C, orange. (Scale bar: 2 nm.)

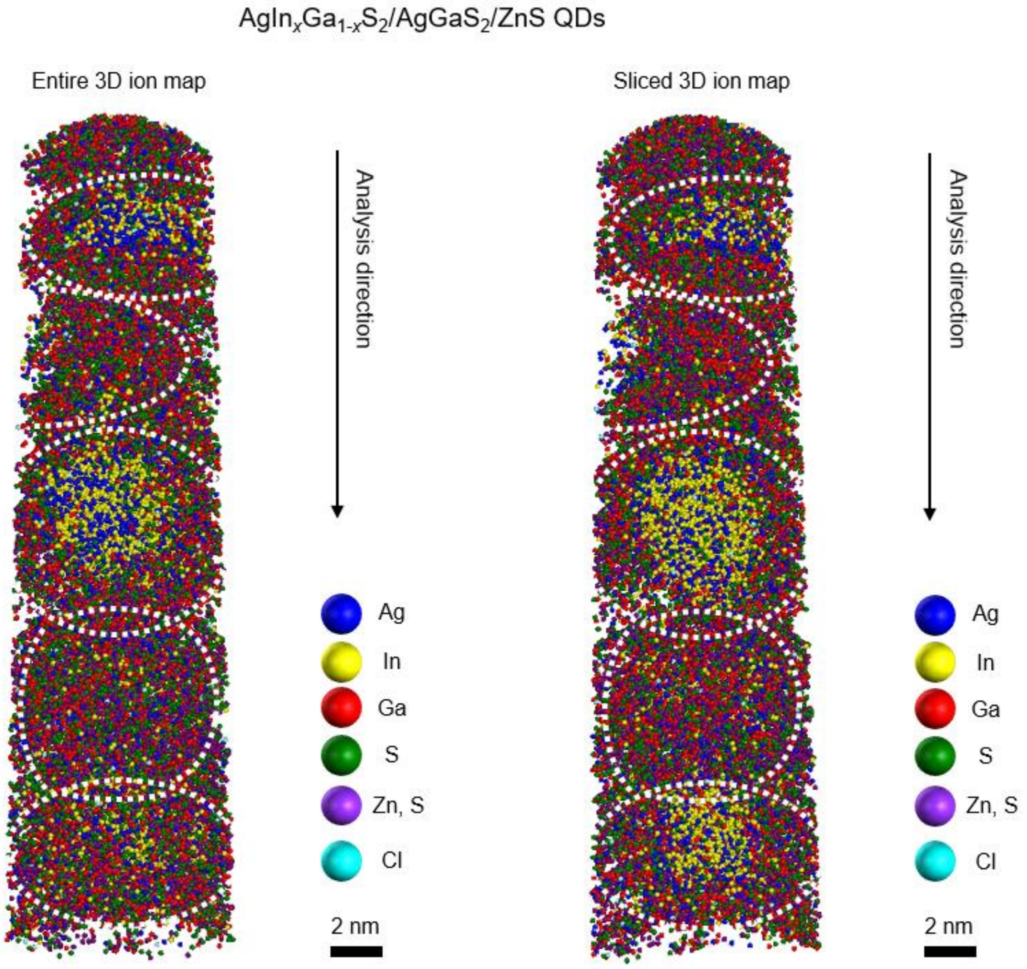

Supplementary Figure 14. APT 3D reconstructions of $AgIn_xGa_{1-x}S_2/AgGaS_2/ZnS$ QDs after the removal of C- and H-related species. C- and H-related ions were excluded from the datasets for visual clarity. For better visualization, the entire 3D ion map (8.5 × 8.5 × 31 nm^3) and a sliced 3D ion map (6 × 8.5 × 31 nm^3) are both shown, with dashed guides indicating the approximate boundaries of individual QDs. Because the QDs are closely packed within the APT specimen, some particles may appear partially overlapped in the projection views. Colors: Ag, blue; In, yellow; Ga, red; S, green; Zn, violet; C, orange; Cl, cyan. (Scale bars: 2 nm.)

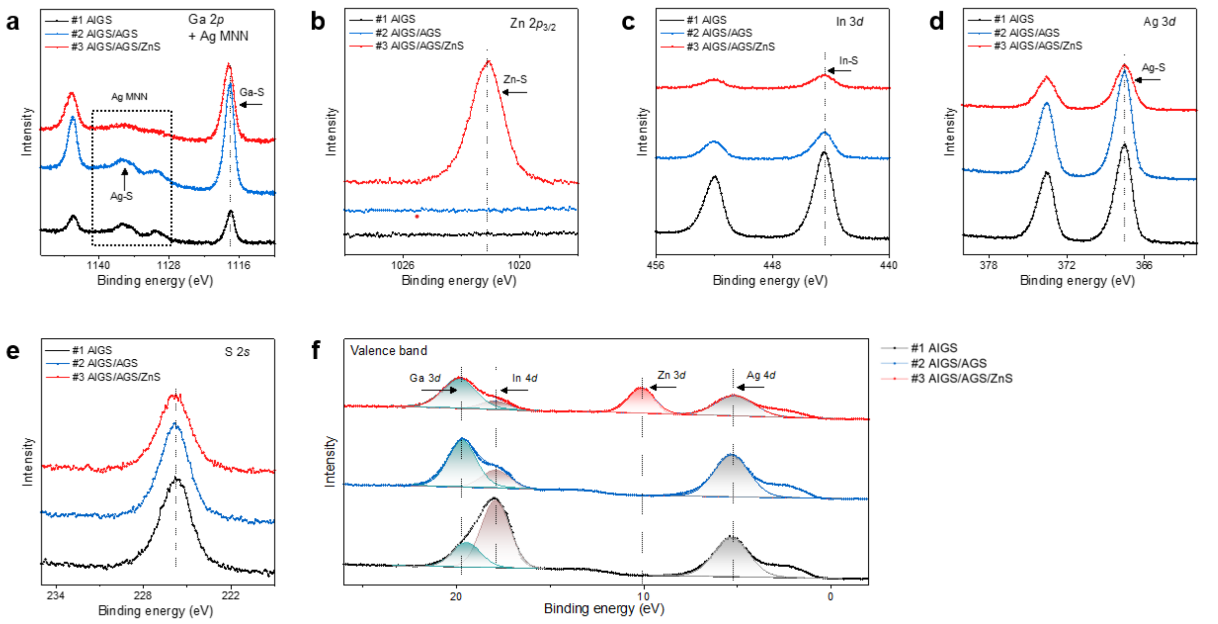

Supplementary Figure 15. XPS analysis results of $AgIn_xGa_{1-x}S_2$-based QDs. XPS spectra of **a** Ga 2*p*, **b** Zn 2*p*$_{2/3}$, **c** In 3d, **d** Ag 3*d*, and **e** S 2*s* core levels acquired from $AgIn_xGa_{1-x}S_2$ (AIGS), $AgIn_xGa_{1-x}S_2/AgGaS_2$ (AIGS/AGS), and $AgIn_xGa_{1-x}S_2/AgGaS_2/ZnS$ (AIGS/AGS/ZnS) QDs using an Al *K*-alpha X-ray source. All QDs contain Ag–S, In–S, Ga–S, and Zn–S chemical bonds, as designed without detectable oxidation. **f** Valence band spectra of AIGS, AIGS/AGS, and AIGS/AGS/ZnS QDs. The Ga-to-Ag ratio is consistently higher in the AIGS/AGS/ZnS QDs than in the AIGS/AGS QDs. The detailed compositions are provided in **Supplementary Table 2.** These results further support the presence of a Ga-containing outer shell, consistent with the $Zn_{1-3/2x}Ga_xS$ shell identified by APT after the ZnS precursor step.

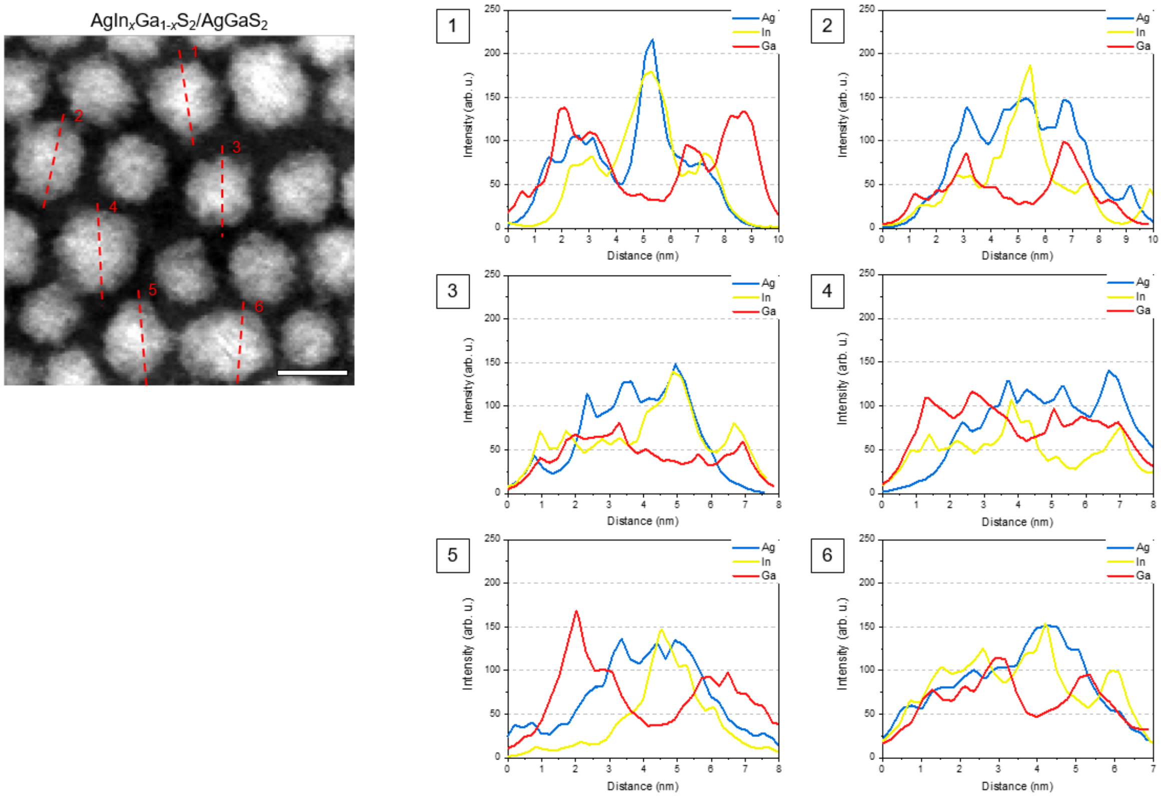

Supplementary Figure 16. Detailed elemental distributions of AgIn$_x$Ga$_{1-x}$S$_2$/AgGaS$_2$ QDs measured by STEM–EDS. STEM image and corresponding EDS line profiles of individual AgIn$_x$Ga$_{1-x}$S$_2$/AgGaS$_2$ QDs. Although the QDs show similar compositional trends, the elemental compositions vary from particle to particle. Owing to the projected nature and limited spatial resolution of STEM–EDS, these data provide qualitative compositional trends rather than a definitive 3D core/shell/shell assignment. (Scale bars: 7 nm.)

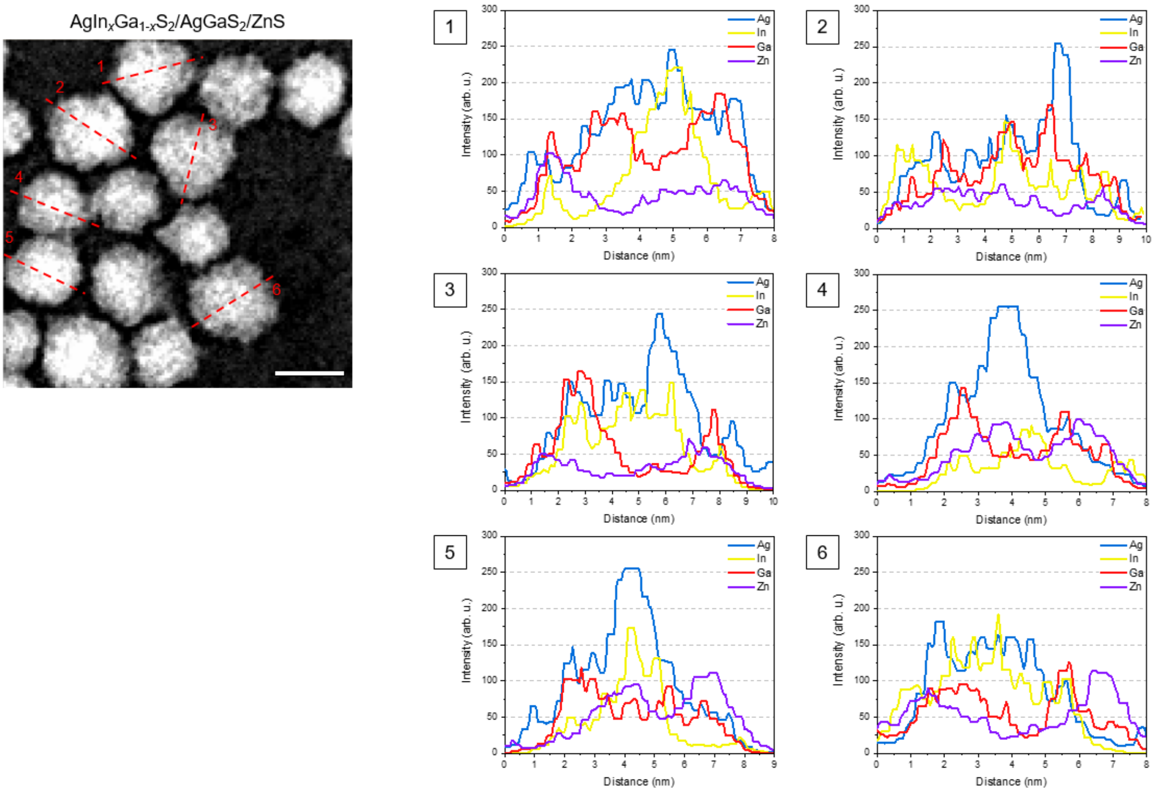

Supplementary Figure 17. Detailed elemental distributions of $AgIn_xGa_{1-x}S_2/AgGaS_2/ZnS$ QDs measured by STEM–EDS. STEM image and corresponding EDS line profiles of individual $AgIn_xGa_{1-x}S_2/AgGaS_2/ZnS$ QDs. Zn is distributed over a broader region than Ag and In, indicating the formation of a Zn-containing outer region. Although the QDs show similar overall compositional tendencies, the elemental compositions vary among individual QDs, indicating particle-to-particle variability. Owing to the projected nature and limited spatial resolution of STEM–EDS, these data provide qualitative compositional trends rather than a definitive 3D core/shell/shell assignment. (Scale bars: 7 nm.)

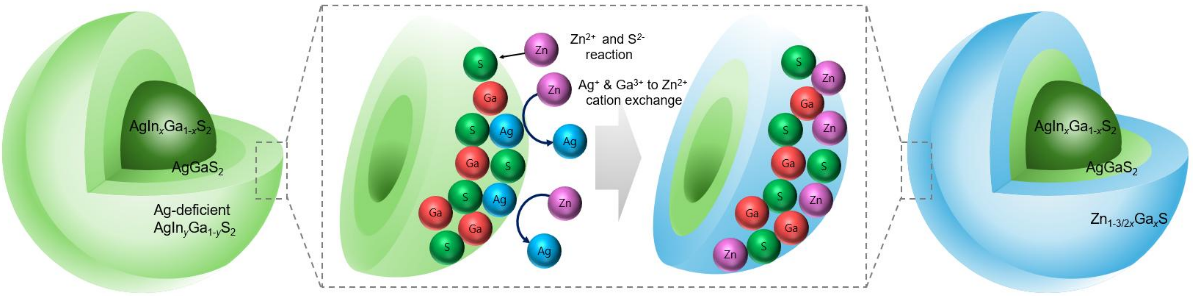

Supplementary Figure 18. Schematic illustrating the hypothesized formation mechanism of the $Zn_{1-3/2x}Ga_xS$ shell after the ZnS precursor step. The outer shell forms through cation exchange, replacing Ag^+ and Ga^{3+} with Zn^{2+}, without changing the size of the QDs, even after the ZnS precursor step for outer shell formation.

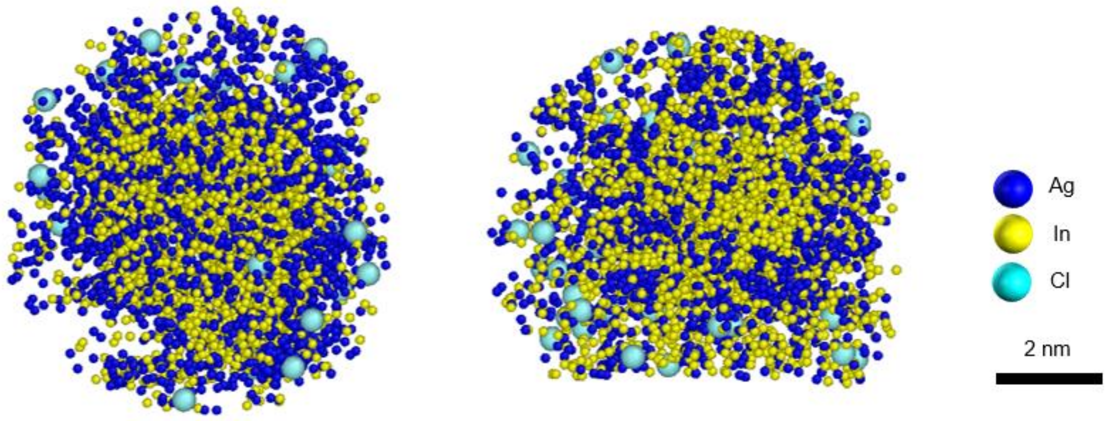

Supplementary Figure 19. 3D Cl map of $AgIn_xGa_{1-x}S_2$/$AgGaS_2$/ZnS QDs. 3D atom maps, showing the distribution of Cl in the outer shell of the QDs. Cl is detected only after the ZnS precursor step. Colors: Ag, blue; In, yellow; Cl, cyan. (Scale bars: 2 nm.)

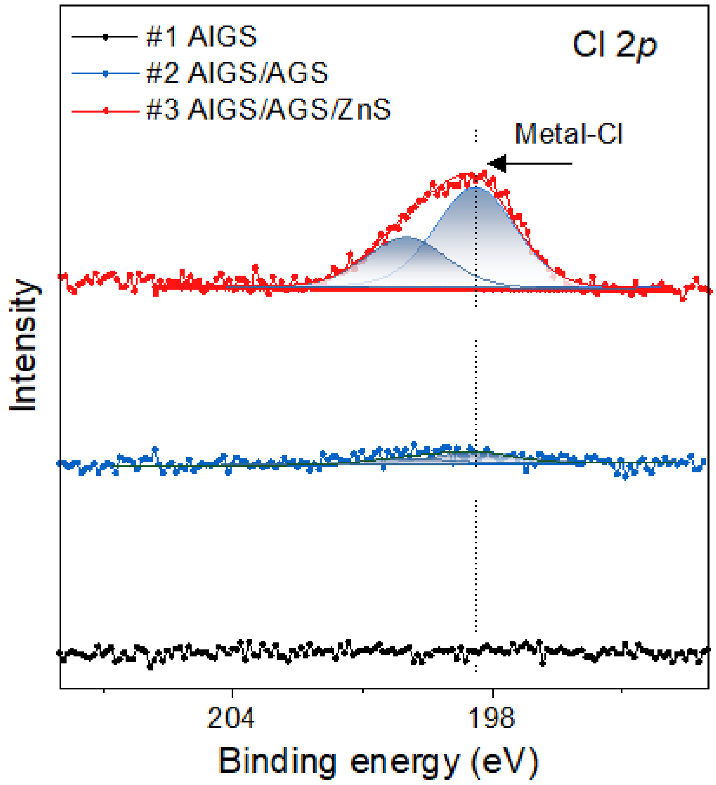

Supplementary Figure 20. XPS Cl 2*p* core-level spectra of $AgIn_xGa_{1-x}S_2$-based QDs. XPS spectra of Cl 2*p* core-level acquired from $AgIn_xGa_{1-x}S_2$ (AIGS), $AgIn_xGa_{1-x}S_2/AgGaS_2$ (AIGS/AGS), and $AgIn_xGa_{1-x}S_2/AgGaS_2/ZnS$ (AIGS/AGS/ZnS) QDs. Cl-related signals are observed only in the AIGS/AGS/ZnS QDs, indicating the presence of metal–Cl chemical states associated with the Zn-based shell introduced during the ZnS precursor step.

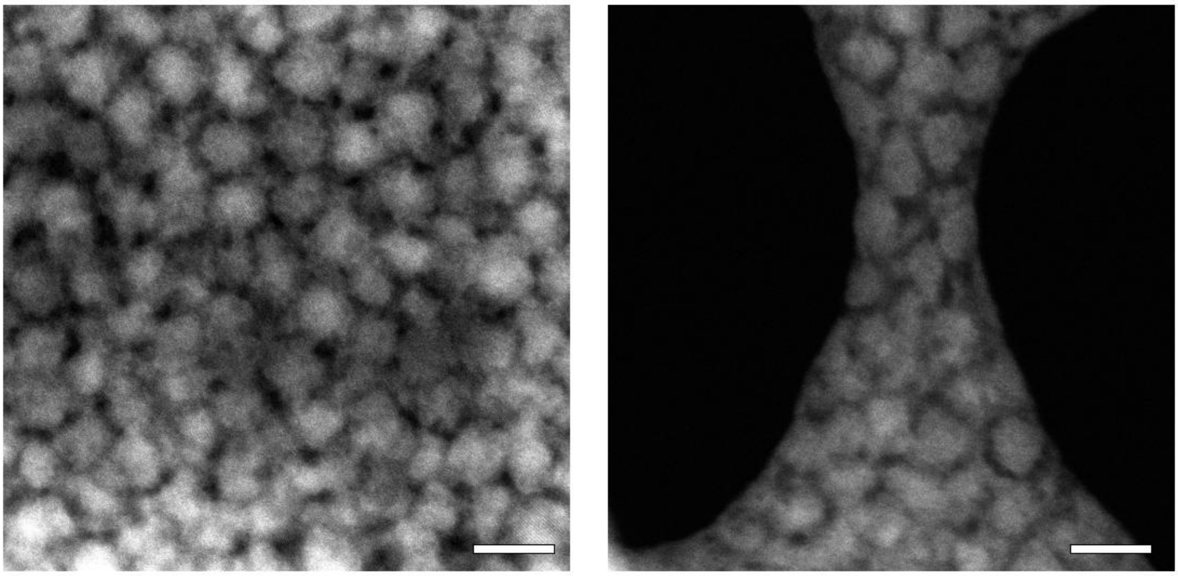

Supplementary Figure 21. Morphology and dispersion of $AgIn_xGa_{1-x}S_2/AgGaS_2/ZnS$ QDs in dried powder form. HAADF–STEM images of $AgIn_xGa_{1-x}S_2/AgGaS_2/ZnS$ QDs in dried powder form, prepared by FIB, showing the representative particle morphology and overall dispersion. The QDs exhibit sizes and shapes comparable to those observed in the solution-dispersed QDs. (Scale bars: 10 nm.)

**Ag precursor amount**	**Ga/Ag** (precursor ratio)	$\lambda_{PL\ max}$ (band edge)	**FWHM** (nm)	**Trap emission area ratio**	**QY**	**QD size measured by TEM** (nm)
Low Ag	29	524	35	5.1	77	6.0
↓	10	520	37	7.8	68	6.8
	6	517	37	12.9	57	6.9
High Ag	4	516	38	21.3	37	7.9

Supplementary Table 1. Dependence of the optical properties and size of $AgIn_xGa_{1-x}S_2/AgGaS_2$ QDs on the Ga/Ag precursor ratio. As the Ag precursor amount increases, the average particle size of the $AgIn_xGa_{1-x}S_2/AgGaS_2$ QDs as measured by TEM increases. Meanwhile, the trap emission increases, and the QY decreases. Owing to the relatively low reactivity of the Ga precursor, increasing the Ag precursor under Ga-rich conditions primarily leads to further growth of the $AgGaS_2$ shell. The trap emission area ratio was defined as the fraction of the integrated PL intensity in the long-wavelength region ($\lambda > \lambda_{PL\ max} + 50$ nm) to the total integrated emission.

Specimen	Atomic composition (%) (XPS)								
	**C** (in C 1*s*)	**O** (in O 1*s*)	**F** (in F 1*s*)	**S** (in S 2*s*)	**Cl** (in Cl 2*p*)	**Zn** (in Zn $2p_{3/2}$)	**Ga** (in Ga $2p_{3/2}$)	**Ag** (in Ag 3*d*)	**In** (in In $3d_{5/2}$)
**AIGS**	76.29	1.91	0	11.54	0	0	1.1	4.46	4.7
**AIGS/AGS**	69.37	2.83	0	14.69	0.23	0	5.16	5.9	1.83
**AIGS/AGS/ZnS**	62.92	6.58	0	13.93	2.07	6.07	4.54	2.85	1.06

Specimen	Atomic composition (%) (XPS)			
	**S** (in S 2*s*)	**Ga** (in Ga $2p_{3/2}$)	**Ag** (in Ag 3*d*)	**In** (in In $3d_{5/2}$)
**AIGS**	52.92	5.07	20.44	21.57
**AIGS/AGS**	53.25	18.71	21.41	6.63
**AIGS/AGS/ZnS**	62.28	20.28	12.73	4.72

Specimen	Atomic composition (%) (XPS)			
	**Ag** (in Ag 4*d*)	**Zn** (in Zn 3*d*)	**In** (in In 4*d*)	**Ga** (in Ga 3*d*)
**AIGS**	36.1	0	37.7	26.2
**AIGS/AGS**	37.1	0	10.0	52.9
**AIGS/AGS/ZnS**	21.3	30.5	5.1	43.1

Supplementary Table 2. Atomic compositions of $AgIn_xGa_{1-x}S_2/AgGaS_2$ (AIGS/AGS) and $AgIn_xGa_{1-x}S_2/AgGaS_2/ZnS$ (AIGS/AGS/ZnS) QDs measured by XPS. The Ga-to-Ag ratio is consistently higher in the AIGS/AGS/ZnS QDs than in the AIGS/AGS QDs, suggesting the formation of a Ga-containing outer shell. This trend is observed both when comparing Ag MNN and Ga 2*p* and when comparing Ag 4*d* and Ga 3*d*, all of which lie in similar binding energy ranges.

Specimen	Atomic ratio (ICP–OES)			
	**Ag/S**	**In/S**	**Ga/S**	**Zn/S**
**AIGS/AGS**	0.40	0.12	0.44	0
**AIGS/AGS/ZnS**	0.13	0.08	0.35	0.46

Supplementary Table 3. Atomic ratios measured of $AgIn_xGa_{1-x}S_2/AgGaS_2$ (AIGS/AGS) and $AgIn_xGa_{1-x}S_2/AgGaS_2/ZnS$ (AIGS/AGS/ZnS) determined by ICP–OES.

Specimen	Atomic ratio (APT)			
	Ag/S	In/S	Ga/S	Zn/S
**AIGS/AGS**	0.43	0.13	0.39	0
**AIGS/AGS/ZnS**	0.15	0.08	0.30	0.48

Supplementary Table 4. Atomic ratios of $AgIn_xGa_{1-x}S_2/AgGaS_2$ (AIGS/AGS) and $AgIn_xGa_{1-x}S_2/AgGaS_2/ZnS$ (AIGS/AGS/ZnS) determined by APT.
